# Genome of *Solanum pimpinellifolium* provides insights into structural variants during tomato breeding

**DOI:** 10.1038/s41467-020-19682-0

**Published:** 2020-11-16

**Authors:** Xin Wang, Lei Gao, Chen Jiao, Stefanos Stravoravdis, Prashant S. Hosmani, Surya Saha, Jing Zhang, Samantha Mainiero, Susan R. Strickler, Carmen Catala, Gregory B. Martin, Lukas A. Mueller, Julia Vrebalov, James J. Giovannoni, Shan Wu, Zhangjun Fei

**Affiliations:** 1 Boyce Thompson Institute, Ithaca, NY 14853 USA; 2 CAS Key Laboratory of Plant Germplasm Enhancement and Specialty Agriculture, Wuhan Botanical Garden, Innovative Academy of Seed Design, Chinese Academy of Sciences, Wuhan, Hubei 430074 China; 3 Plant Pathology and Plant-Microbe Biology Section, School of Integrative Plant Science, Cornell University, Ithaca, NY 14853 USA; 4 US Department of Agriculture-Agricultural Research Service, Robert W. Holley Center for Agriculture and Health, Ithaca, NY 14853 USA

**Keywords:** Comparative genomics, Structural variation, Natural variation in plants, Plant domestication

## Abstract

*Solanum pimpinellifolium* (SP) is the wild progenitor of cultivated tomato. Because of its remarkable stress tolerance and intense flavor, SP has been used as an important germplasm donor in modern tomato breeding. Here, we present a high-quality chromosome-scale genome sequence of SP LA2093. Genome comparison identifies more than 92,000 structural variants (SVs) between LA2093 and the modern cultivar, Heinz 1706. Genotyping these SVs in ~600 representative tomato accessions identifies alleles under selection during tomato domestication, improvement and modern breeding, and discovers numerous SVs overlapping genes known to regulate important breeding traits such as fruit weight and lycopene content. Expression quantitative trait locus (eQTL) analysis detects hotspots harboring master regulators controlling important fruit quality traits, including cuticular wax accumulation and flavonoid biosynthesis, and SVs contributing to these complex regulatory networks. The LA2093 genome sequence and the identified SVs provide rich resources for future research and biodiversity-based breeding.

## Abstract (web summary)

*Solanum pimpinellifolium* (SP) is the progenitor of cultivated tomato and an important germplasm. Here, the authors assemble SP genome, identify structural variants (SVs) by comparing with modern cultivar, reveal SVs associated with important breeding traits, and detect SVs harboring master regulators of fruit quality traits.

## Introduction

Tomato (*Solanum lycopersicum*) is the world’s leading vegetable crop with a total production of 182 million tons and a worth over US $60 billion in 2018 (http://www.fao.org/faostat). *S. pimpinellifolium* (SP) carrying red, small, and round fruits is the wild progenitor of the cultivated tomato. It was domesticated in South America to give rise to *S. lycopersicum* var. *cerasiforme* (SLC), which was later improved into the big-fruited tomato *S. lycopersicum* var. *lycopersicum* (SLL) in Mesoamerica^1,2^. The fact that SP can freely hybridize with SLL has enabled the incorporation of SP alleles into modern tomato cultivars to improve disease resistance, abiotic stress tolerance, and other fruit quality traits^3^. Due to the importance of SP, draft genome assemblies of two accessions, LA1589 (ref. ^4^) and LA0480 (ref. ^5^), have been generated using Illumina short-read sequencing technology. Although these assemblies provided some essential genomic information for SP, they are highly fragmented and incomplete, limiting their applications as robust references in tomato breeding and research. Recently, a chromosome-scale assembly of an SP accession, BGV006775, was generated using Nanopore long reads, providing a valuable resource for the Solanaceae community^6^.

Genomic structural variants (SVs), including insertions/deletions (indels), inversions, and duplications, are the causative genetic variants for many domestication traits of crops^7^. Empirical cases in tomato include a ~294-kb inversion at the *fas* locus leading to enlarged fruits^8^, a 1.4-kb deletion in the *CSR* gene resulting in an increased fruit weight^9^, a partial deletion of the *LNK2* gene leading to the circadian period lengthening in cultivated tomatoes^10^, and tandem duplications at both *sb1* and *sb3* that suppress the excessive inflorescence branching^11,12^. Whole-genome SNP data have been employed to reveal the impact of human selection on the tomato genome^13^ and reconstruct tomato domestication history^14^. Recently, researchers have started to explore the distribution of SVs in tomato accessions and their population dynamics^12^. Identification of SVs between SP and SLL, studying their evolutionary dynamics in different tomato populations and investigating their regulatory roles in the context of gene expression can provide critical insights into the contribution of SVs to important agronomic traits in tomato domestication and breeding.

In this study, we present a high-quality chromosome-scale genome sequence of an SP accession, LA2093, assembled from PacBio long reads combined with Hi-C chromatin interaction maps. LA2093 harbors many desirable traits and has served as the donor parent of a recombinant inbred line (RIL) population that has been widely used for mapping disease resistance and fruit quality traits^15–19^. We further identify SVs between the genomes of LA2093 and cultivar Heinz 1706 through direct genome comparison combined with PacBio long read mapping, and genotype this reference set of SVs in ~600 tomato accessions representing SP, SLC, and heirloom and modern SLL to determine their population dynamics. We also employ expression quantitative trait locus (eQTL) mapping to explore the roles of SVs in regulating gene expression and identify eQTL hotspots and potential master regulators controlling important fruit traits.

## Results

### Sequencing and assembly of the *S. pimpinellifolium* genome

**Fig. 1:**
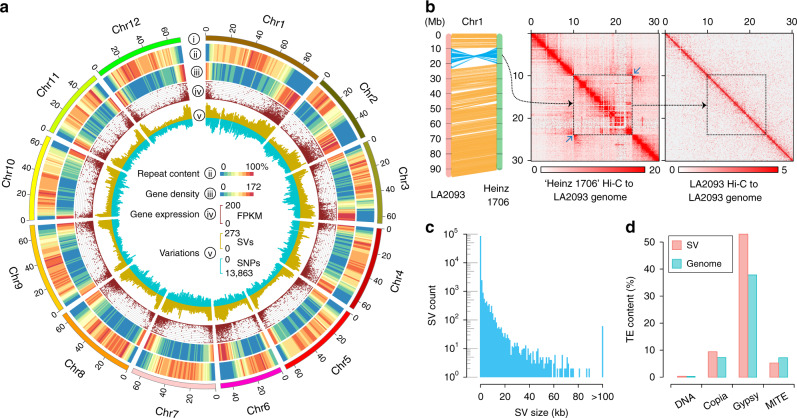
Genomic landscape of *S. pimpinellifolium* LA2093 and structural variants identified between LA2093 and Heinz 1706. **a** Features of the LA2093 genome. (i) Ideogram of the 12 chromosomes in Mb scale. (ii) Repeat content (% nucleotides per Mb); (iii) gene density (number of genes per Mb). (iv) Gene expression (FPKM). (v) Densities of SVs (structural variants) (outer) and SNPs (inner) in comparison to Heinz 1706 (number of SVs and SNPs per Mb). **b** Alignment of chromosome 1 between LA2093 and Heinz 1706. The color intensity in Hi-C heatmaps represents the number of links between two 100-kb windows. The inversion shown in blue (left) is supported by high-density contacts pointed by the two blue arrows in Hi-C heatmaps generated from Heinz 1706 Hi-C reads aligned to the LA2093 genome (middle), while no corresponding contract is found in the LA3093 Hi-C heatmap (right). **c** Distribution of SV sizes. **d** Contents of different categories of transposable elements in SV regions and the whole-genome of LA2093.

We assembled the genome of LA2093 using PacBio long reads and Hi-C chromatin contact information. A total of 96 Gb of PacBio sequences with an N50 read length of 22.3 kb was generated, covering approximately 103× of the LA2093 genome with an estimated size of 923 Mb (Supplementary Fig. 1). The PacBio reads were de novo assembled into contigs, followed by polishing with both PacBio and Illumina reads. This resulted in an assembly of 453 contigs with a total length of 807.6 Mb and an N50 length of 10.9 Mb (Supplementary Data 1). A total of 166 million Hi-C read pairs were generated for constructing chromatin interaction maps. These Hi-C contact maps, together with the synteny with the Heinz 1706 genome^20^ (version 4.0) and the genetic map constructed using the NC EBR-1 × LA2093 RIL population^16^, were used to scaffold the assembled contigs. Finally, 385 contigs with a total length of ~800 Mb, accounting for 99.0% of the assembly, were clustered into 12 pseudomolecules (Fig. 1a). The Hi-C heatmap (Supplementary Fig. 2) and the good collinearity between the pseudomolecules and the genetic map (Supplementary Fig. 3) supported the chromosome-scale structure of the assembly. BUSCO^21^ assessment indicated that about 97.8% of the core conserved plant genes were found complete in the LA2093 assembly (Supplementary Data 1). Further evaluation using Merqury^22^ revealed a consensus quality score (QV) of 46 for the LA2093 assembly (Supplementary Table 1). Collectively, these results indicated that the LA2093 genome assembly is of high quality, comparable to or better than the BGV006775 assembly^6^ but with substantially improved contiguity and completeness compared to the two previously reported *S. pimpinellifolium* genome assemblies^4,5^ (Supplementary Table 1 and Supplementary Data 1). A total of 544.3 Mb repetitive sequences (67.3%) were identified in the LA2093 assembly with the *gypsy* retrotransposon being the most abundant repeat family (37.8%; Supplementary Table 2). The genome was predicted to harbor 35,761 protein-coding genes, of which 35,535 (99.4%) were supported by RNA-Seq data, and/or homologs in the NCBI non-redundant protein database.

### Genomic SVs between LA2093 and Heinz 1706

Alignment of the genome sequences of LA2093 and Heinz 1706 (version 4.0) showed good collinearity between the two reference genomes (Supplementary Fig. 4). Despite the high collinearity, 28 inversions ranging from 483 bp to 13.9 Mb were identified distributing across all 12 chromosomes (Supplementary Data 2), which were further supported by PacBio reads and/or Hi-C maps (Fig. 1b and Supplementary Fig. 5). Together these inversions harbored approximately 800 genes (861 in LA2093 and 733 in Heinz 1706), of which a large portion (457) were annotated with functions related to disease resistance and response to abiotic stress (Supplementary Data 3).

In addition to inversions, indels between LA2093 and Heinz 1706 genomes were detected by direct comparison of the two high-quality assemblies combined with mapping of PacBio long reads to the genomes. A total of 92,523 indels, ranging from 10 bp to 2.4 Mb, were identified (Fig. 1a, c and Data 4). As expected, the majority of the indels were relatively short with 82.8% <100 bp and only 0.1% >100 kb (Fig. 1c). Approximately 52.9% of these indel sequences were *gypsy*-like retrotransposons, compared to 37.8% of the entire genome, while the contents of other types of transposable elements were similar between the indel regions and the whole-genome (Fig. 1d), suggesting that indels occurred more frequently in genome regions occupied by *gypsy*-like retrotransposons.

SVs in gene body and promoter regions can impact gene functions and expression. Only 14.8% of identified indels overlapped with gene body or promoter (defined here as 3-kb upstream of gene body) regions, notably lower than the proportion in the whole-genome (27.7%), implying a functional constraint against these indels on coding or regulatory regions (Supplementary Table 3). More than half of the predicted genes in LA2093 (21,875 out of 35,761) and Heinz 1706 (19,590 out of 34,689) had at least one indel in their gene body or promoter regions, with 2,419 in LA2093 and 1,710 in Heinz 1706 having indels in their coding sequences (CDS) (Supplementary Fig. 6). Genes affected by the identified indels were enriched with those involved in response to stimulus, reproduction, signal transduction, and primary and secondary metabolic processes (Supplementary Fig. 7), suggesting that these indels may contribute to the differences in disease resistance and fruit quality traits between the wild and cultivated tomatoes. We detected several SVs known to underlie tomato domestication traits, such as the 1.4-kb deletion and 22-bp insertion in the *CSR* gene leading to increased fruit weight^9^, the 7-kb deletion in the *LNK2* gene responsible for the circadian period lengthening in cultivated tomatoes^10^, the 4-kb substitution in the promoter of the *TomLoxC* gene, which contributes to fruit flavor^17,23,24^, and the 85-bp deletion in the promoter of the *ENO* gene, which regulates floral meristem activity^25^ (Supplementary Data 5).

### Selection of SVs in tomato domestication and breeding

De novo detection of SVs based on short read alignments to a reference genome is subject to a relatively high rate of both false negatives and false positives^26,27^. Therefore, the SVs identified between LA2093 and Heinz 1706 through direct genome comparison and PacBio long read mapping provided a valuable set of reference SVs that could be used to investigate the roles of SVs in tomato domestication and breeding. The 92,523 indels and 28 inversions were genotyped in 597 tomato accessions, including 51 SP, 6 *S. cheesmaniae* and *S. galapagense* (SCG), 228 SLC, 226 heirloom, 52 modern and 34 other cultivars (Supplementary Data 6). To estimate the accuracy of SV genotyping using short read mapping to the reference SVs, we generated 31.1 Gb of Nanopore long read sequences for an SP accession, LA1589, and aligned the long reads to the LA2093 and Heinz 1706 genomes for SV calling. About 96.2% of the genotypes determined using the LA1589 short reads were confirmed by the Nanopore long read mapping method. Genotyping the SVs in the two reference accessions using Illumina short reads further supported a high specificity of our SV genotyping (Supplementary Table 4). Phylogenetic and population structure analyses using the SVs clearly separated the SP and SCG groups from the heirloom and modern groups with SLC being the intermediate group between the wild and cultivated accessions. A similar pattern was observed using whole-genome SNPs (Supplementary Fig. 8), further assuring the high specificity of our identified SVs. Seven accessions positioned into unexpected species groups were excluded from downstream analyses (Supplementary Data 6).

**Fig. 2:**
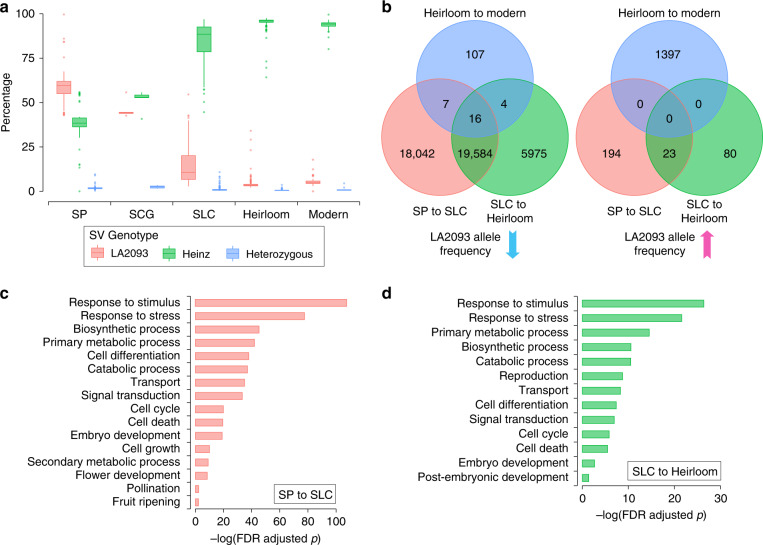
SVs under selection during tomato domestication and breeding. **a** Percentages of SVs with different genotypes in each accession of different groups. The numbers of accessions are 50, 6, 224, 225, and 51 in SP, SCG, SLC, Heirloom, and Modern groups, respectively. For each box plot, the lower and upper bounds of the box indicate the first and third quartiles, respectively, and the center line indicates the median. The whisker represents 1.5× interquartile range of the lower or upper quartile. **b** Venn diagrams of selected SVs during domestication (SP to SLC), improvement (SLC to heirloom) and modern breeding (heirloom to modern). **c**, **d** GO terms enriched in genes affected by SVs selected during domestication (**c**) and improvement (**d**). Enriched GO terms were identified using two-tailed Fisher’s exact test, adjusted for multiple comparisons. Source data underlying Fig. 2a are provided as a Source data file.

SV allele frequency changes among different tomato populations are a result of evolutionary events, such as selection of desirable traits, reduced population size, and introgression from ancestral groups. To identify SVs under selection during tomato domestication and breeding, we investigated SV allele frequency changes from SP to SLC for domestication, from SLC to SLL heirlooms for improvement, and from heirlooms to modern elite lines for modern breeding. In the SP population, SV loci with the homozygous LA2093 alleles were prevalent, making up an average of 58.4% of the SVs in each accession, while only 39.4% SV loci had the homozygous Heinz 1706 genotypes (Fig. 2a). After domestication and improvement, the frequencies of Heinz 1706 alleles increased to 84.3% and 95.3% in SLC and heirloom tomatoes, respectively, and then slightly decreased to 93.6% in modern lines (Fig. 2a). These findings implied substantial genetic diversity loss imposed by domestication, especially the loss of the SP-specific alleles. The allele frequencies of 38,367 SVs were significantly changed between different tomato populations (Supplementary Data 4). During domestication, the LA2093 allele frequencies of 17 inversions and 37,632 indels were significantly lower in SLC than in SP, while only 217 indels had the LA2093 allele frequencies significantly higher in SLC (Fig. 2b). These selected indels could affect 14,189 genes in LA2093 and 12,264 in Heinz 1706. In the improvement process, only 103 indels had higher LA2093 allele frequencies in heirlooms than in SLC, and 25,579 SVs (13 inversions and 25,566 indels) displayed significantly lower LA2093 allele frequencies in heirlooms (Fig. 2b), which collectively could affect 9530 and 7870 genes in LA2093 and Heinz 1706, respectively. The enriched functions were shared between genes affected by the SVs selected during domestication and improvement, including stress and stimulus response, biosynthesis, cell differentiation, embryo development, pollination, and reproduction processes (Fig. 2c, d). These results demonstrated a common selection preference for the Heinz 1706 alleles in tomato domestication and improvement. It is worth noting that, despite the continuous loss of wild species alleles from SP to SLC and SLL, 1397 SVs exhibited significantly higher LA2093 allele frequencies in modern lines than in heirlooms (Fig. 2b). This could be related to the re-introduction of agriculturally favorable alleles from wild accessions into modern SLL lines.

The nucleotide diversity in the modern group was similar to that of the heirlooms but lower than that in SLC and SP for most genomic regions. However, a substantially higher nucleotide diversity level was observed in the modern group compared to the heirlooms on chromosomes 4, 5, and 11, consistent with the higher *F*_ST_ values between the two populations on these chromosomes (Supplementary Fig. 9a, b). Concordantly, introgressions of genomic regions from SP to modern cultivars were identified in all these three chromosomes (Supplementary Fig. 9c), which are largely consistent with those identified in a recent study^12^. The introgressions on chromosome 4 contained a cold tolerance QTL^28^, on chromosome 5 carried several known QTLs controlling soluble solid content (SSC) in fruit^13^, and on chromosome 11 included an important disease resistant genes, *Sm*^29^ (Supplementary Fig. 9d). These results imply that SP introgressions in the modern cultivars might be acquired through contemporary breeding to introduce abiotic and biotic stress tolerance and favorable flavor traits.

### SV selection associated with breeding traits

Tomato fruit phenotypes have changed dramatically during domestication and improvement. Investigating population dynamics of SVs potentially affecting the expression or functions of genes controlling important horticultural traits can improve our understanding of the impacts of human selection on these genes and provide potential targets for breeding. Fruit size enlargement is a major domestication syndrome in tomato. Several SVs have been identified to be associated with fruit weight, including the 1.4-kb deletion in *CSR* and the 85-bp deletion in the promoter of *ENO*, both of which underlie larger fruit size^9,25^. Consistent with that reported in Mu et al.^9^, we found that the allele frequency of the 1.4-kb deletion in *CSR* was 1.0% and 11.5% in SP and SLC, respectively, and became largely fixed in SLL with 90.2% in heirlooms and 94.1% in modern lines (Supplementary Data 5). The 85-bp deletion in the *ENO* promoter had a frequency of 54.7% in SP, 90.8% in SLC, 94.0% in heirloom and 96.8% in modern accessions. Interestingly, we identified an additional 257-bp insertion in *CSR* spanning the 5′ UTR and the CDS, which had a frequency of 38.6% in SP, 77.9% in SLC, 98.3% in heirloom and 97.7% in modern accessions, and another 57-bp deletion in the *ENO* promoter, whose allele frequencies in SP, SLC, SLL heirloom and modern were 22.0%, 83.5%, 92.3%, and 94.6%, respectively. Furthermore, indels were detected for additional fruit weight genes, including a 23-bp insertion in the promoter of *RRA3a*, which had an allele frequency pattern that was suggestive of selection during domestication (Supplementary Data 5).

**Fig. 3:**
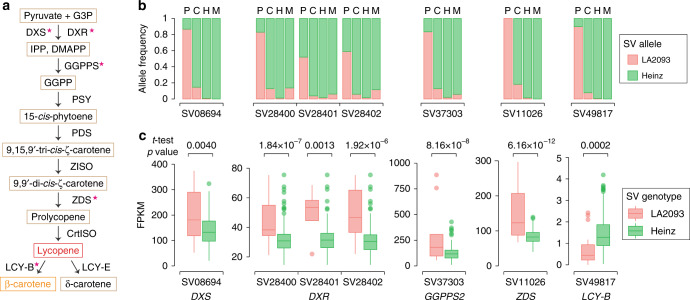
Selected SVs affecting the expression of lycopene metabolism genes. **a** Lycopene biosynthesis and degradation pathway in tomato. Stars indicate enzyme-coding genes having selected SVs associated with significantly different gene expression levels between the two alleles (two-tailed Student’s *t*-test *p* value <0.01). **b** Allele frequencies of selected SVs in SP (P), SLC (C), heirloom (H) and modern (M) populations. **c** Gene expression levels in tomato accessions carrying the homozygous LA2093 and Heinz 1706 alleles, respectively, of the selected SVs. For each SV from left to right, the numbers of accessions with homozygous LA2093 alleles are 11, 20, 4, 10, 9, 9, and 18, and those with homozygous Heinz alleles 162, 210, 131, 129, 164, 155, and 244, respectively. Two-tailed Student’s *t*-test was performed to compare expression levels of each gene between the accessions with homozygous LA2093 and with Heinz alleles for each SV. For each box plot, the lower and upper bounds of the box indicate the first and third quartiles, respectively, and the center line indicates the median. The whisker represents 1.5× interquartile range of the lower or upper quartile. G3P, glyceraldehyde 3-phosphate; IPP, isopentenyl diphosphate; DMAPP, dimethylallyl diphosphate; GGPP, geranylgeranyl diphosphate; DXS, 1-deoxy-d-xylulose 5-phosphate synthase; DXR, 1-deoxy-D-xylulose-5-phosphate reductoisomerase; GGPPS, geranylgeranyl pyrophosphate synthase; PSY, phytoene synthase; ZISO, ζ-carotene isomerase; ZDS, ζ-carotene desaturase; CrtISO, carotene isomerase; LCY-B, lycopene β-cyclase; LCY-E, lycopene ε-cyclase. Source data underlying Fig. 3c are provided as a Source data file.

Fruit lycopene levels seem to have decreased upon the origin of SLC and then have largely remained in SLL^14^. We identified indels in genes that control lycopene biosynthesis and cyclization, of which the Heinz 1706 allele frequencies were significantly increased from SP to SLC and reached near fixation in heirlooms (Supplementary Data 5). These Heinz 1706 alleles, mostly deletions in promoters, were associated with decreased expression of *1‐deoxy‐D‐xylulose 5‐phosphate synthase* (*DXS*), *1-deoxy-D-xylulose-5-phosphate reductoisomerase* (*DXR*), *geranylgeranyl pyrophosphate synthase 2* (*GGPPS2*) and *ζ-carotene desaturase* (*ZDS*), and higher expression of *LCY-B* in the ripening fruit (Fig. 3), suggesting that these mutations might cause reduced lycopene levels in SLC and SLL by downregulating lycopene biosynthetic genes, and promoting lycopene degradation through upregulation of *LCY-B*. Interestingly, we found a recovery of certain LA2093 alleles in the elite tomato lines, including the ones potentially associated with higher expression of *DXR* and *GGPPS2* (Supplementary Data 5).

Fruit ripening regulation is of great interest to tomato researchers and breeders. Tomato FUL1, FUL2, and TAGL1 play key roles in controlling fruit ripening by interacting with RIN^30,31^. Here, we identified a 15-bp in-frame deletion (SV28464) in the CDS of *FUL1* of Heinz 1706, which had frequencies of 1.0%, 75.5%, and 99.0% in SP, SLC, and heirlooms, respectively. Intriguingly, *FUL1* was expressed at a considerably higher level in ripe fruits of the elite tomato line, NC EBR-1, carrying the derived deletion allele, than in LA2093 that carries the non-deletion allele (Supplementary Fig. 10a). Consistently, accessions with homozygous *FUL1* deletion allele expressed the gene at a higher level in the orange-stage fruit than those with the non-deletion allele (Supplementary Fig. 10b). Together, these results suggested that the *FUL1* deletion allele was associated with the higher *FUL1* expression and positively selected during domestication. *FUL2* of Heinz 1706 carried three SVs [an 87-bp deletion (SV28464), a 22-bp deletion (SV28465) and a 15-bp insertion (SV28466)] in its promoter. SV28464 had frequencies of 46.3%, 86.7% and 98.4%, SV28465 24.3%, 88.9% and 99.4%, and SV28466 14.3%, 85.1% and 96.2% in SP, SLC and heirlooms, respectively (Supplementary Data 5), suggesting their positive selection during tomato domestication and/or improvement. All these three SVs were associated with the reduced expression of *FUL2* in the orange-stage fruit (Supplementary Fig. 10b). The seemingly opposite effects of the selected alleles on the expression of *FUL1* and *FUL2* in orange-stage fruit could be related to the different spatial and temporal expression patterns of *FUL1* and *FUL2* in ripening fruit and their specific developmental functions^31,32^. In addition, one 38-bp deletion (SV58448) and one 28-bp insertion (SV58449) were found in the last intron of *TAGL1* of Heinz 1706, of which SV58448 had frequencies of 1.1%, 55.4% and 85.2% and SV58449 32.3%, 63.0% and 84.1% in SP, SLC and heirlooms, respectively (Supplementary Data 5). In addition, the 28-bp deletion was found to be associated with higher expression of *TAGL1* in cultivated tomatoes (Supplementary Fig. 10b).

Wild species have been used as a source for improving SSC in cultivated tomatoes^33,34^. Three well-characterized genes that contribute to fruit SSC, include *SUCR* controlling sucrose accumulation^35^, and *Lin5* and *Agp-L1* regulating hexose content^34^. We found a 13-bp insertion (SV26359) in the first intron of *SUCR* associated with lower expression of the gene (Supplementary Fig. 11), whose frequencies were 13.3% in SP, 86.5% in SLC, 99.4% in heirloom and fixed in modern accessions. LA2093 carried a 15-bp in-frame deletion in *Lin5* (SV67476), resulting in a 5-aa deletion at one amino acid upstream of the critical amino acid for sucrose binding^34^. The frequency of the non-deletion allele was substantially increased during domestication (33.7% in SP, 90.9% in SLC, 98.9% in heirloom and fixed in modern accessions). For *AgpL1*, we identified an 18-bp deletion in the second intron (SV12806) and a 33-bp deletion in the promoter (SV12807), both having significantly increased allele frequencies in SLC and SLL compared to SP (37.5% in SP, 85.2% in SLC and 96.2% in heirloom for the 18-bp deletion, and 32.7% in SP, 85.2% in SLC and 96.3% in heirloom for the 33-bp deletion; Supplementary Data 5). Domestication traits, including high fruit yield, increased fruit:leaf ratio and determinant habit, are found to be negatively correlated with high SSC^35^. Although the Heinz 1706 alleles of these SSC genes were nearly fixed in the modern tomatoes, possibly a result of hitchhiking with domestication and improvement traits, the LA2093 alleles identified here offer an opportunity to improve SSC in the elite lines.

Incorporating alleles from wild species into SLL has been a strategy in tomato breeding to improve disease resistance. We identified selected SVs in a number of well-studied disease resistance genes (Supplementary Data 5). Further functional characterization of these SVs may open a door to recover the disease-resistance traits in cultivated tomatoes. We also identified selected SVs for genes involving in hormonal regulation, flower, inflorescence, seed and leaf development, as well metabolite biosynthesis (Supplementary Data 5), which would be associated with the dramatic changes of morphotype and metabolite diversity during the long history of tomato breeding.

### Genome-wide mapping of eQTLs

**Fig. 4:**
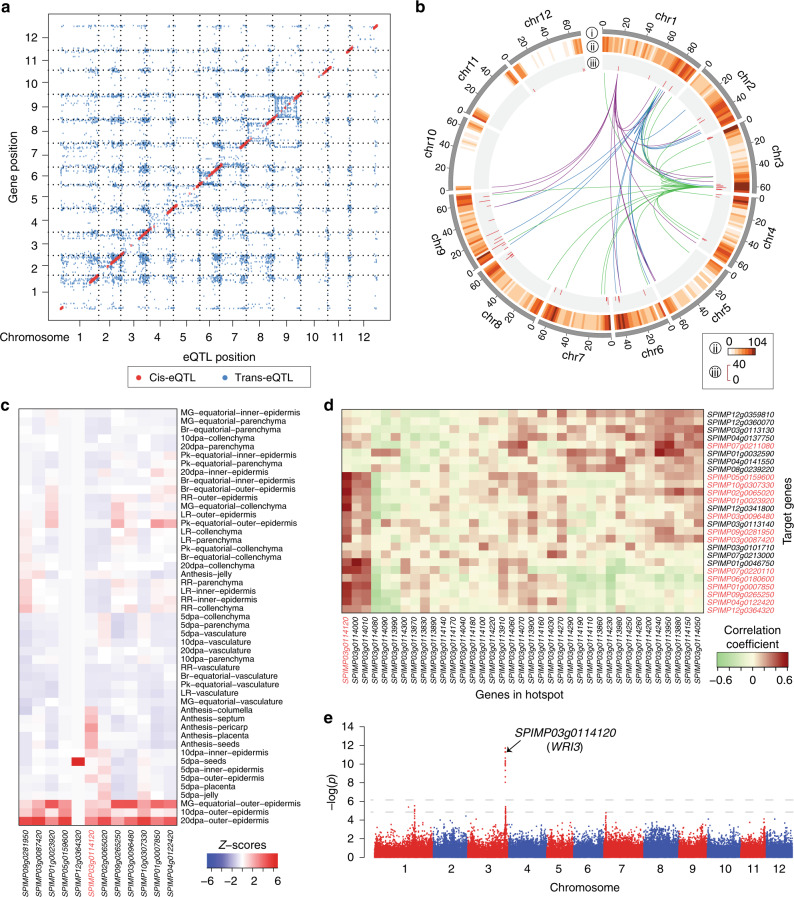
Genome-wide mapping of eQTLs. **a** Positions of eQTLs identified in the genome. **b** Trans-eQTL hotspots. The outermost circle displays ideograms of the 12 tomato chromosomes. The second circle shows the number of target genes for all eQTLs in each 2-Mb window. The third circle shows the number of target genes of each trans-eQTL hotspot. The innermost circle shows the links between three interesting eQTL hotspots and their target genes. Links between the three hotspots harboring the master regulators, A20/AN1 zinc finger protein, MYB12, and WRI3, respectively, and their target genes are depicted in purple, blue and green, respectively. **c** Expression profiles of WRI3-targeted lipid biosynthetic genes in different tomato tissues. The *WRI3* gene (*SPIMP03g0114120*) is highlighted in red. **d** Correlation coefficients between the expression levels of genes in the *WRI3*-hotspot and target genes. *WRI3* and the target lipid biosynthetic genes are highlighted in red. **e** Manhattan plot of eQTLs associated with the *WRI3* expression. The horizontal dashed lines correspond to the Bonferroni-corrected significance thresholds at α = 0.05 and α = 1.

To explore the roles of SVs in gene expression regulation, we performed eQTL analysis using the published orange-stage fruit transcriptome data^36^. A total of 46,848 SVs in 10,789 eQTL regions were identified to be significantly associated with the expression of 5595 genes, including 2708 (25.1%) cis- and 8081 (74.9%) trans-eQTLs (Fig. 4a and Supplementary Data 7**)**. The cis-eQTLs were more significantly associated with the gene expression and thus explained more expression variation than the trans-eQTLs (Supplementary Fig. 12a, b). The lead SVs of most cis-eQTLs were located near the start or end of coding regions (Supplementary Fig. 12c), indicating the important regulatory roles of sequences in these regions on gene expression.

The genomic distribution of trans-eQTLs was assessed to identify eQTL hotspots underlying the major regulatory variations across the tomato accessions. A total of 48 hotspots regulating the expression of 554 genes, as well as the potential master regulator within each hotspot, were identified (Fig. 4b and Supplementary Data 8). One hotspot located on chromosome 1 around 76.5 Mb (*P* = 1.68 × 10^−17^) contained the *MYB12* gene, which encodes a key regulator of flavonoid biosynthesis^36–38^. Among the 18 genes whose expression was regulated by this hotspot, seven encoded flavonoid biosynthetic enzymes (Supplementary Data 9), including chalcone synthase and flavonoid 3′-hydroxylase, which have been proved to be the direct targets of MYB12 (ref. ^39^). Interestingly, another hotspot (Chr1:18800715-19894697; *P* = 2 × 10^−11^) was found to affect the expression of the flavonoid biosynthetic genes regulated by the MYB12 hotspot. This hotspot contained an A20/AN1 zinc finger gene, whose homolog in poplar mediates flavonoid biosynthesis^40^. Furthermore, a total of 17 additional eQTLs were detected for 17 flavonoid biosynthetic genes (Supplementary Fig. 13 and Supplementary Data 10), among which 14 genes had trans-eQTLs and three had cis-eQTLs. Further study of these eQTLs will likely advance our knowledge on the gene regulatory network of flavonoid biosynthesis.

A major hotspot was found on chromosome 3 with 26 target genes, 14 of which were associated with lipid metabolism (Supplementary Data 11). An AP2/ERF transcription factor orthologous to Arabidopsis *WRI3* (ref. ^41^), was identified as the master regulator targeting 17 genes, including those encoding pyruvate kinase, pyruvate dehydrogenase, acetyl-coenzyme A carboxylase, carboxyl transferase and acyl carrier proteins (Supplementary Data 11). Co-expression of the tomato *WRI3* with lipid metabolism genes in the epidermis of developing fruit^42^ suggested its potential function in tissue-specific regulation of lipid biosynthesis, thus contributing to fruit cuticular wax accumulation (Fig. 4c). The expression of *WRI3* was positively correlated with that of all its target genes (Fig. 4d), suggesting its positive regulatory role. Furthermore, eQTL analysis identified two SVs, a 11-bp insertion in the sixth intron and a 77-bp deletion located in the promoter of *WRI3*, that were significantly associated (*P* value = 1.93e−12) with its expression (Fig. 4e**;** Supplementary Fig. 14 and Supplementary Data 5). Altogether, these results suggested that *WRI3* could be a key regulator of fruit outer epidermis lipid deposition.

## Discussion

*S. pimpinellifolium* is thought to be the wild progenitor of cultivated tomatoes and offers desirable agronomic traits for breeding. The currently available chromosomal-scale SP assemblies were built based on the Heinz 1706 reference^6,12^. In this study, by utilizing PacBio long read sequencing, Hi-C chromatin contact maps and genetic maps, we provide the high-quality pseudomolecules of the SP genome that complements the existing tomato Heinz 1706 reference and serves as the foundation for exploring the genetic potential of SP and studying the genome evolution from wild to cultivated tomato under human selection. Explicit comparison between the genomes of LA2093 and Heinz 1706 enabled us to discover megabase-scale large SVs that could not be detected previously due to limitation of sequencing read length^12^. By investigating the frequency changes of wild alleles at the identified SV loci between LA2093 and Heinz 1706 in a large collection of ~600 tomato accessions representing SP, SLC, heirloom, and modern SLL, we not only revealed the population dynamics of known causal SVs underlying important traits during tomato domestication, improvement, and modern breeding, but also identified numerous candidate SVs in or near well-characterized genes controlling horticultural traits. The population dynamics of these SVs provide useful information for future determination of their likelihood of being the causal genetic variants. Furthermore, these SVs may be used as potential targets for future breeding programs to improve fruit quality and stress tolerance.

The LA2093 alleles of most selected SVs had significantly reduced frequencies after domestication and improvement. The massive loss of these wild alleles in cultivated tomatoes could be related to the large selective sweeps associated with domestication and improvement^13^. Similar to the finding of metabolic genes hitchhiked with *fw11.3* (ref. ^36^), linkage drag also contributed to the recovery of many LA2093 alleles in modern cultivars on chromosomes 4, 5, and 11, likely associated with recent intentional introduction of disease resistance, stress tolerance, and favorable fruit quality traits into cultivars from wild accessions. Large structural variations complicate the cloning of genes underlying target traits and introgression of wild alleles in breeding without bringing along unwanted hitchhikers. Our study provides ample information that can be used to facilitate the recovery of wild beneficial alleles into modern elite cultivars.

eQTL analysis helped to unravel the hierarchical regulatory relationships among genes and provided insights into the effects of SVs on gene expression. In addition to confirming the central role of MYB12 in regulating flavonoid biosynthesis in tomato fruit, we identified additional eQTLs containing potential regulatory genes and SVs contributing to the complex regulatory network of flavonoid biosynthesis. Tomato fruit cuticular lipids provide a barrier against water loss and microbial infection^43,44^. An AP2/ERF transcription factor gene, *WRI3*, may serve as a master regulator that controls the tissue-specific expression of key lipid biosynthetic enzyme genes in tomato fruit epidermis. These results increased our knowledge of the regulatory mechanism involved in fruit cuticular lipid accumulation, which could be applied to the development of crops with improved post-harvest performance.

## Methods

### Library construction and sequencing

Plants of the *Solanum pimpinellifolium* accession LA2093 were grown in the greenhouse at Boyce Thompson Institute in Ithaca, New York, with a 16-h light period at 20 °C (night) to 25 °C (day).

A PacBio SMRT library was constructed from the high molecular weight DNA following the standard SMRTbell library preparation protocol and sequenced on a PacBio Sequel platform using the 2.0 chemistry (PacBio). Meantime, an Illumina paired-end library with insert size of ~450 bp was constructed using the Illumina Genomic DNA Sample Preparation kit following the manufacturer’s instructions (Illumina), and a Hi-C library was prepared following the proximo Hi-C plant protocol (Phase Genomics). Both the Illumina paired-end and the Hi-C libraries were sequenced on an Illumina NextSeq 500 platform with the paired-end mode and read length of 150 bp.

Nanopore sequences were generated for the SP accession LA1589 using the method described in Mazo-Molina et al.^45^ and used for validation of SV genotyping. Briefly, Nanopore libraries were constructed from the HMW DNA of LA1589 using the Ligation Sequencing Kit (SQK-LSK109) and sequenced on the MinION R9 flow cells for 48 h. Basecalling was performed using Guppy (v3.1.5).

Total RNA was extracted from immature, mature green and red ripe fruits using the QIAGEN RNeasy Plant Mini Kit (QIAGEN). Strand-specific RNA-Seq libraries were constructed using the protocol described in Zhong et al.^46^ and sequenced on an Illumina HiSeq 2000 platform (Illumina). Three biological replicates were performed for each sample.

### De novo assembly of the LA2093 genome

Raw PacBio reads were error corrected and assembled into contigs using CANU^47^ (v1.7.1) with default parameters except that ‘OvlMerThreshold’ and ‘corOutCoverage’ were set to 500 and 200, respectively. PacBio reads were then aligned to the contigs and based on the alignments errors in the assembled contigs were corrected using the Arrow program implemented in SMRT-link-5.1 (PacBio). Furthermore, the Illumina paired-end reads were processed to remove adaptor and low-quality sequences using Trimmomatic^48^ (v0.36). The cleaned Illumina reads were aligned to the contigs using BWA-MEM^49^ (v0.7.17) with default parameters, and based on the alignments two rounds of iterative error corrections were performed using Pilon^50^ (v1.22) with parameters ‘–fix bases–diploid’. The final error-corrected contigs were then compared against the NCBI non‐redundant nucleotide database, and those with more than 95% of their length similar to sequences of organelles (mitochondrion or chloroplast) or microorganisms (bacteria/fungi/viruses), were considered contaminants and discarded. The redundans pipeline^51^ (v0.14a) was then used to remove redundancies in the assembled contigs with parameters ‘--identity 0.99 --overlap 0.97’.

To scaffold the assembled contigs, Illumina reads from the Hi-C library were processed with Trimmomatic^48^ (v0.36) to remove adaptor and low-quality sequences. The cleaned Hi-C reads were aligned to the assembled contigs and the alignments were filtered using the Arima-HiC mapping pipeline (https://github.com/ArimaGenomics/mapping_pipeline). Based on the alignments, the contigs were clustered into pseudomolecules using SALSA^52^ (v2.2) with parameters ‘-e GATC -i 3’. Furthermore, contigs of LA2093 were also assembled into pseudomolecules by comparing them with the Heinz1706 reference genome^20^ (version 4.0) using RaGOO^6^ (v1.1). Inconsistencies between pseudomolecules constructed using the Hi-C data and those using the synteny information with the Heinz1706 genome were identified. The mis-joined scaffolds were manually corrected based on the Hi-C contact information, genome synteny information, and a genetic map constructed from a recombinant inbred line (RIL) population with LA2093 as one of the parents^16^, resulting a consensus set of LA2093 pseudomolecules. Finally, the genetic map was also used to validate the final consensus set of LA2093 pseudomolecules using ALLMAPS^53^ (v0.8.12). Inconsistencies between the LA2093 pseudomolecules and genetic maps were also manually checked and the accuracy of the LA2093 pseudomolecules was further validated using PacBio read alignment information.

### Annotation of the LA2093 genome assembly

MITE-Hunter^54^ (v11-2011) and LTRharvest^55^ (v1.5.10) were used to de novo identify miniature inverted-repeat transposable elements (MITEs) and long terminal repeats (LTRs), respectively, in the assembled LA2093 genome. The LA2093 genome was masked using RepeatMasker (v4.0.8; http://www.repeatmasker.org/) with the identified MITEs and LTRs, and the unmasked genome sequences were then analyzed using RepeatModeler (v1.0.11; http://www.repeatmasker.org/RepeatModeler.html) to build a de novo repeat library. The final repeat library was obtained by combining the MITEs, LTRs and the de novo repeat library, and subsequently used to screen the LA2093 genome for repeat sequences using RepeatMasker. The identified repeat sequences were classified using the RepeatClassifier program of RepeatModeler.

Protein-coding genes were predicted from the repeat-masked LA2093 genome. LA2093 RNA-Seq reads generated in this study and from a previous study^16^ were processed to trim low-quality and adapter sequences using Trimmomatic^48^ (v0.36). The cleaned high-quality RNA-Seq reads were aligned to the assembled genome using HISAT2 (ref. ^56^) (v2.1) with default parameters. Transcripts were assembled from the read alignments using StringTie^57^ (v1.3.3b). The complete coding sequences (CDS) were predicted from the assembled transcripts using the PASA pipeline^58^ (v2.3.3). Ab initio gene predictions were performed using BRAKER^59^ (v2.0.4), GeneMark-ET^60^ (v4.61), and SNAP^61^ (v2006-07-28). Protein sequences from the Swiss-Prot database and from Heinz 1706, *S. pennellii*, pepper and potato were used as protein homology evidence. Finally, the high-confidence gene models in the LA2093 genome were predicted using the Maker pipeline^62^ (v2.31.10) by integrating ab initio predictions, transcript mapping and protein homology evidence.

### Detection of SVs and SNPs between reference genomes

To identity SVs between genomes of LA2093 and Heinz 1706 (SL4.0), the two genomes were first aligned using Minimap2 (ref. ^63^) (v2.17) with the parameter ‘-ax asm5’. The resulting alignments were analyzed using Assemblytics^64^ (v1.1) for SV identification. The identified SVs spanning or close (<50 bp) to gap regions in either of the two genomes were excluded.

SVs were also identified using pbsv (v2.2; https://github.com/PacificBiosciences/pbsv) and SVIM^65^ (v1.2.0) by aligning LA2093 PacBio reads to the Heinz1706 genome and Heinz 1706 PacBio reads to the LA2093 genome. The identified SVs spanning gap regions in the genomes were discarded. For large inversions identified through direct comparison of the two genomes, the two breakpoints of a candidate inversion were identified based on the genome alignment results. To confirm the breakpoints, the supported split reads were extracted from the results of pbsv and SVIM. Inversions were kept if more than 90% of total reads spanning the breakpoint were split reads or they were detected by both pbsv and SVIM. For the remaining SVs, the 5-kb flanking sequences of each SV were extracted from the reference genome, and then blasted against the query genome. The blast hits were then compared to the unique alignments between LA2093 and Heinz 1706 genomes identified by Assemblytics^64^. An SV was kept if the following criteria were met: (1) the blast hits of the two flank sequences of the SV (alignment length >50 bp, identity >90%, e-value <1e−10) were in the expected region on the query genome; and (2) the distance between the two blast hits was largely consistent with the SV size estimated by PacBio read mapping. For insertions, we required that the difference in SV size determined by PacBio read mapping and distance between the two blast hits was smaller than 20% of the estimated SV size. For deletions, the allowed gaps or overlaps between the two blast hits of flanking regions should be smaller than 3 bp. Repeat expansions/contractions and tandem expansions/contractions detected by Assemblytics^64^ were converted into one or more simple indels if the precise breakpoints were defined using the SVs identified by pbsv. SVs identified by both Assemblytics and pbsv were combined if they overlapped with each other (>50% in each). GO term enrichment analysis for the SV-related genes was performed using the Fisher’s exact test in the Blast2GO suite^66^ (v1.3.11) with a cutoff of adjusted *P* value <0.05.

SNPs between the two genomes were identified by comparing LA2093 and Heinz 1706 genomes using MUMmer4 (ref. ^67^) (v4.0.0). The uniquely aligned fragments were used to identify SNPs with the show-snp tool in the MUMmer4 package.

### SV and SNP genotyping in the tomato population

Genome resequencing data of 725 tomato accessions reported in Gao et al.^17^ were downloaded from the NCBI SRA database (Supplementary Data 6). The downloaded raw Illumina sequences from each accession were first processed to consolidated duplicated read pairs, which were defined as those having identical bases in the first 90 bp (for 100-bp reads) or 100 bp (for 150-bp reads) of both left and right reads, into unique read pairs. The resulting reads were processed to trim adaptor and low-quality sequences using Trimmomatic^48^ (v0.36).

The high-quality reference SVs identified between the LA2093 and Heinz1706 genomes were genotyped in the 725 tomato accessions. The cleaned Illumina reads from each accession were aligned to the LA2093 and Heinz1706 genomes, respectively, using BWA-MEM^49^ (v0.7.17) allowing no more than 3% mismatches. For each SV in each accession, reads aligned to regions spanning the breakpoints of the SV in both LA2093 and Heinz1706 genomes were extracted and checked. For inversions, only split reads were used as evidence and each breakpoint was supported by at least three split reads. For indels with breakpoints supported by less than three split reads, the read coverage in the deleted regions were further checked. For an indel, we required that <50% of the deleted region was covered by reads with 2× depth, while >50% of at least one of the flanking regions with the same length of the deleted region was covered. Based on the split read and read depth information, SVs in a particular accession were classified as the LA2093 genotype (same as in LA2093), the Heinz1706 genotype (same as in Heinz 1706), heterozygous (containing both LA2093 and Heinz1706 alleles), or undetermined (genotypes that could not be determined due to insufficient read mapping information). Accessions with less than 40% of SVs genotyped were excluded, leaving 597 tomato accessions kept for the downstream analyses.

To evaluate the accuracy of our SV genotyping using short reads, the reference SVs were also genotyped in LA1589 using Nanopore long reads. The Nanopore reads were first self-corrected using NECAT^68^ (v0.0.1) and then further corrected using Illumina reads with fmlrc^69^ (v0.1.2). The corrected long reads were aligned to the LA2093 and Heinz1706 genomes, respectively, using Minimap2 (ref. ^63^) (v2.17). Based on the alignments, SVs were called using SVIM^65^ (v1.2.0) and compared with those genotyped using short reads in LA1589 to estimate the accuracy of SV genotyping.

Illumina reads aligned to the LA2093 genome were used for SNP calling. First, the duplicated alignments were marked using Picard (v2.3; http://broadinstitute.github.io/picard/), with the parameter ‘OPTICAL_DUPLICATE_PIXEL_DISTANCE = 250’. The ‘HaplotypeCaller’ function of GATK^70^ (v3.8) was then used to generate a GVCF file for each accession with parameters ‘--genotyping_mode DISCOVERY --max_alternate_alleles 3 --read_filter OverclippedRead’, followed by the population variant calling using the function ‘GenotypeGVCFs’ with default parameters. Hard filtering was applied to the raw variant calling set, with parameters ‘QD < 2.0 | | FS > 60.0 | | MQ < 40.0 | | MQRankSum < −12.5 | | ReadPosRankSum < −8.0’. Sites with at least 50% accessions genotyped and minor allele frequency (MAF) ≥ 0.03 and overlapping with the identified SNPs from the alignments of LA2093 and Heinz1706 genomes were kept.

### Population genomic analyses

Maximum-likelihood phylogenetic trees were constructed for the tomato accessions using the full SV dataset and SNPs at four-fold degenerated sites, respectively, using IQ-TREE^71^ (v1.6.8) with 1000 bootstraps. Population structure was investigated using FastStructure^72^ with default parameters. To analyze the selection of SVs during domestication, improvement and modern breeding, the frequency of each allele of a particular SV in each group was calculated. Significance of the difference of the frequencies between two compared groups was determined using Fisher’s exact test. The resulting raw *P* values of SVs were then corrected using the Bonferroni method and SVs with corrected *P* values <0.001 were defined as those under selection. The nucleotide diversity and population differentiation index (*F*_ST_) across the genome were calculated using VCFtools^73^ (v0.1.16) based on the SVs and SNPs. *F*_ST_ values were calculated in each 1000-kb window with a step size of 250 kb.

Putative introgressions between two groups were identified using a likelihood ratio test^74^ with the SV datasets. For each SV site in an accession of the heirloom or modern group, the percentage of accessions sharing this SV genotype was first derived in each of the two groups, then the ratio of the percentage of accessions sharing the genotype in the group this specific accession belonging to that of accessions in the SP group was calculated. The average ratio for all SV sites in each of the 1000-kb windows with a step size of 250 kb were obtained. Regions with ratios of 0.9 or less and containing two or more SVs were defined as introgressions.

### RNA-Seq expression and eQTL analysis

Raw RNA-Seq data of 399 accessions reported in Zhu et al.^36^ and of LA2093 and NC EBR-1 at four different stages of fruit development reported in Gao et al.^17^ were downloaded from the NCBI SRA database under the accessions PRJNA396272 and PRJNA659593, respectively. Raw sequencing reads were processed to trim low-quality and adaptor sequences using Trimmomatic^48^. The cleaned reads were aligned to the LA2093 genome using HISAT2 (ref. ^56^) (v2.1.0). Based on the alignments, raw read counts were derived for each gene and normalized to fragments per kilobase transcripts per million mapped fragments (FPKM). For eQTL analysis, expression data from 305 out of the 399 accessions that had SV data in this study were used. Genes with a median FPKM value of zero were excluded from the downstream analysis. Principal component analysis was performed based on the FPKM values, and nine accessions with FPKM values greater than 2.5 standard deviations from the mean in any of the first three principal components were excluded from the downstream analysis. To obtain a normal distribution of expression values for each gene, FPKM values of each gene were further normalized using the quantile-quantile normalization (qqnorm) function in R. To identify the hidden and confounding factors in the expression data, the normal quantile transformed expression values were processed using the probabilistic estimation of expression residuals (PEER) method^75^. The first 20 factors were selected as additional covariates in the genome-wide association studies. The Balding-Nichols kinship matrix constructed using EMMAX^76^ (v20120210) with all SNPs and SVs was used to correct population structure. The missing genotypes in the raw biallelic SV dataset were imputed using the k-nearest neighbor (KNN) algorithm implemented in the fillGenotype software^77^. In order to obtain the optimal imputation accuracy and filling rate, the accession with fewest missing genotypes in SP, SLC and Heirloom were selected and 10%, 20%, and 30% SV sites were randomly masked as missing genotypes for imputing. The imputations were performed using the fillGenotype with the combinations of the parameters: *w* = 20, 30, 50, 65 or 80), *p* = −3, −5, −7 or −9, k = 3, 5, 7, or 9), and *r* = 0.65, 0.7, 0.75, and 0.8. The optimal combination of parameters (*w* = 80, *k* = 3, *p* = −7, *r* = 0.8) was selected after comparing the filling rate and imputation accuracy of each combination of the parameters (Supplementary Table 5). Only biallelic imputed SVs with minor allele frequency ≥ 1% and missing data rate ≤ 40% (a total of 71,684 SVs) were used for eQTL analysis. Genome-wide associations of transformed expression were estimated using the linear mixed model implemented in EMMAX^76^ (v20120210). The Bonferroni test criteria at α = 0.05 and α = 1 were used as thresholds for significant and suggestive associations between variations and traits (expression), respectively, as described in Li et al.^78^. In this study, the Bonferroni-corrected thresholds for the *P* values were 6.97 × 10^−7^ at α = 0.05 and 1.40 × 10^−5^ at α = 1, with corresponding −log10(*P*) values of 6.15 and 4.85, respectively.

Linkage disequilibrium (LD) decay was measured by calculating correlation coefficients (*r*^2^) for all pairs of SVs within 10 Mb using PopLDdecay^79^ (v3.27) with parameters ‘-MaxDist 500 -MAF 0.05 -Het 0.88 -Miss 0.999’. The stable *r*^2^ value was considered as the background level of LD. The background level of LD and physical distance were 0.205 and 1.94 Mb in this study, respectively. SVs within close vicinity to each other and associated with the expression of the same gene were grouped into an eQTL block, represented by the most significant SV (the lead SV) in the block. Two adjacent SVs were grouped into one region if the *r*^2^ > 0.205 and physical distance <1.94 Mb, and regions with at least three significant SVs were considered as candidate eQTL blocks. eQTLs were considered cis if they located within 50 kb of transcription start sites or transcription stop sites of the corresponding genes; otherwise, the eQTLs were considered trans.

eQTL hotspots were identified using the hot_scan software^80^ with a window size of 50 kb and an adjusted *P*-value less than 0.05. Pairwise Pearson correlation coefficients between the target genes and genes located inside the eQTL hotspots were calculated using the FPKM values. The candidate master regulator was identified for each eQTL hotspot using the iterative group analysis (iGA) approach^81^.

### Reporting summary

Further information on research design is available in the Nature Research Reporting Summary linked to this article.

## Supplementary information

Supplementary Information

Peer Review

Reporting Summary

Description of Additional Supplementary Files

Supplementary Data 1

Supplementary Data 2

Supplementary Data 3

Supplementary Data 4

Supplementary Data 5

Supplementary Data 6

Supplementary Data 7

Supplementary Data 8

Supplementary Data 9

Supplementary Data 10

Supplementary Data 11

## Data Availability

Data supporting the findings of this work are available within the paper and its Supplementary information files. A reporting summary for this Article is available as a Supplementary information file. The datasets and plant materials generated and analyzed during the current study are available from the corresponding author upon request. Raw genome and transcriptome sequencing reads have been deposited into the NCBI BioProject database under accession PRJNA607731. The genome assembly of *Solanum pimpinellifolium* LA2093 has been deposited at DDBJ/ENA/GenBank under the accession JAAONP000000000. The genome sequence of LA2093 and the associated annotations are also available at Sol Genomics Network (https://solgenomics.net/). The SV genotyping data in the 597 tomato accessions is available via Figshare [https://figshare.com/articles/dataset/tomato_SV_matrix/12922151].  Source data are provided with this paper.

## Source data

Source data

## Code availability

Computational pipelines related to SV detection and genotyping are available at GitHub [https://github.com/GaoLei-bio/SV].
