# Supplementary material for: Genome of Solanum pimpinellifolium provides insights into structural variants during tomato breeding
Source: Nat Commun. 2020 Nov 16;11:5817. doi: 10.1038/s41467-020-19682-0 (PMC7670462; doi:10.1038/s41467-020-19682-0)
Supplement: Supplementary file 1 — Supplementary Information [file 41467_2020_19682_MOESM1_ESM.pdf]

Genome of *Solanum pimpinellifolium* provides insights into structural variants during tomato breeding

Wang and Gao *et al.*

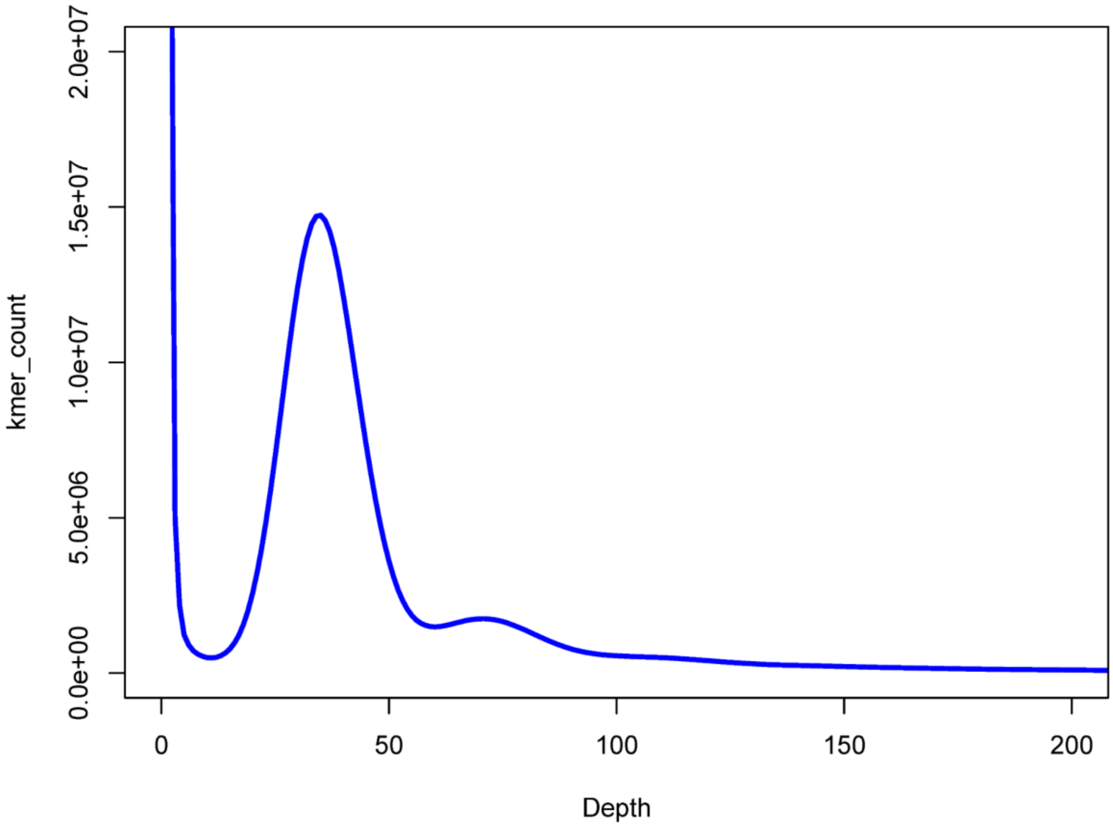

Supplementary Figure 1. 17-mer depth distribution of the LA2093 Illumina genomic reads. Genome size of LA2093 was estimated based on the formula: Total number of Kmers/Position of peak depth = 3,229,3481,394/ 35 = 922,670,896 bp.

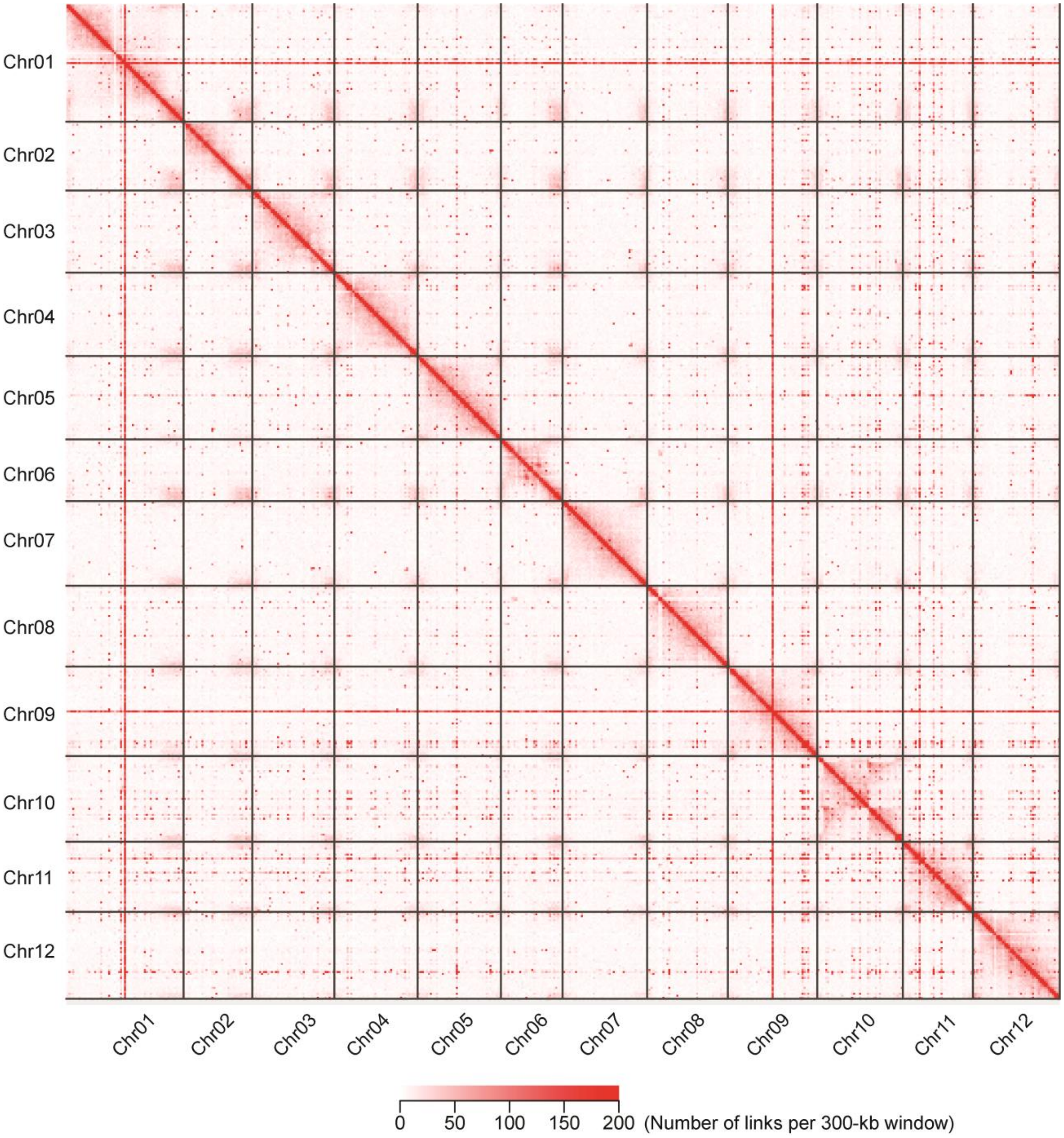

Supplementary Figure 2. Hi-C interaction heatmap of the LA2093 genome.

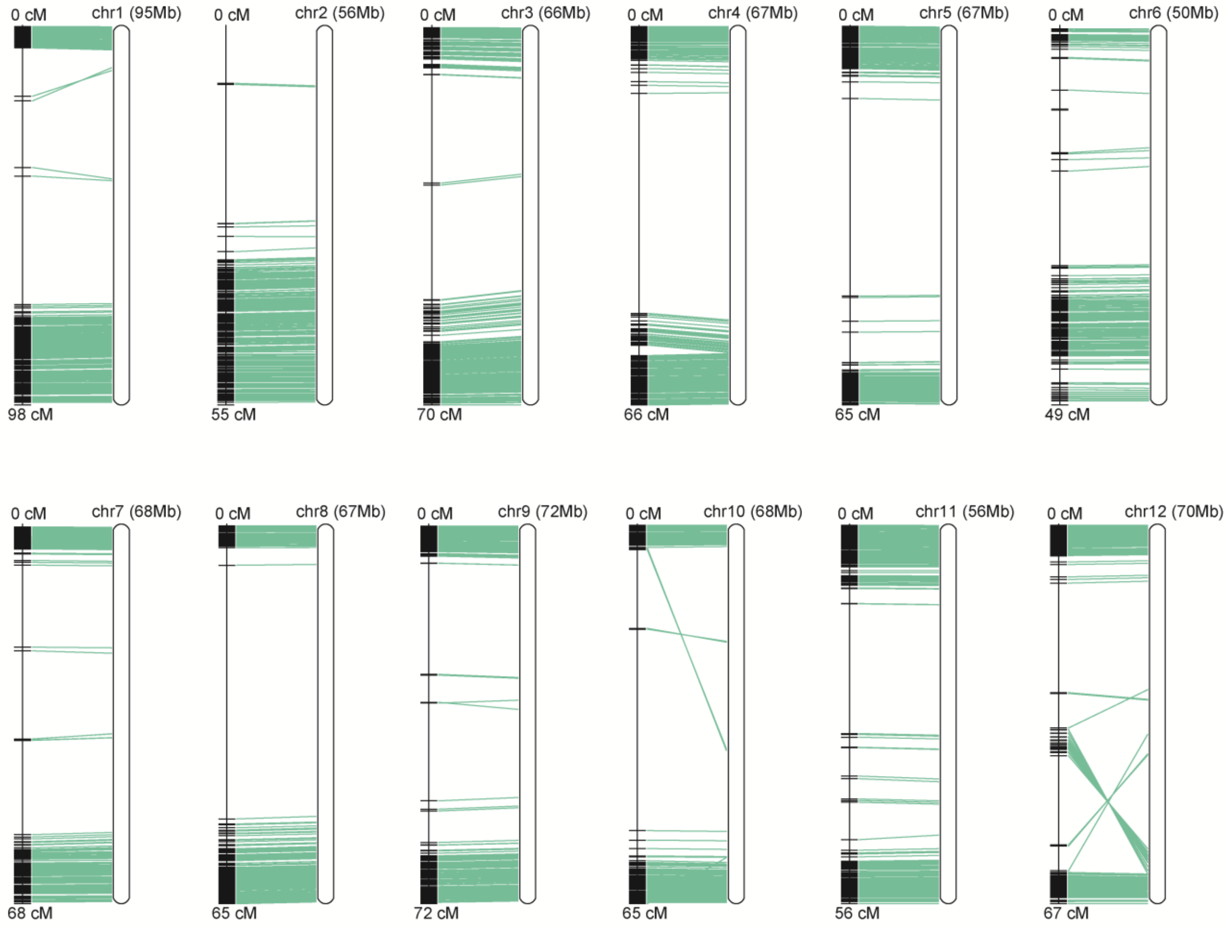

Supplementary Figure 3. Collinearity between the NC EBR-1 × LA2093 genetic map and LA2093 pseudomolecules. Inconsistencies between the genetic map and the assembly were manually checked with Hi-C map and PacBio read mapping information to ensure the accuracy of the genome assembly.

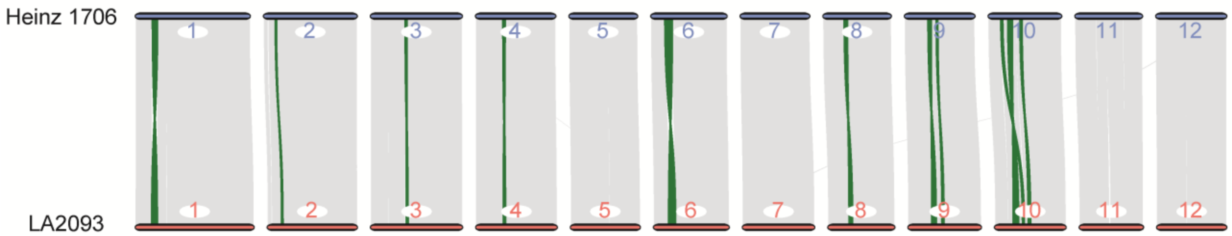

Supplementary Figure 4. Collinearity between genomes of LA2093 and Heinz1706. The inversions are shown in green.

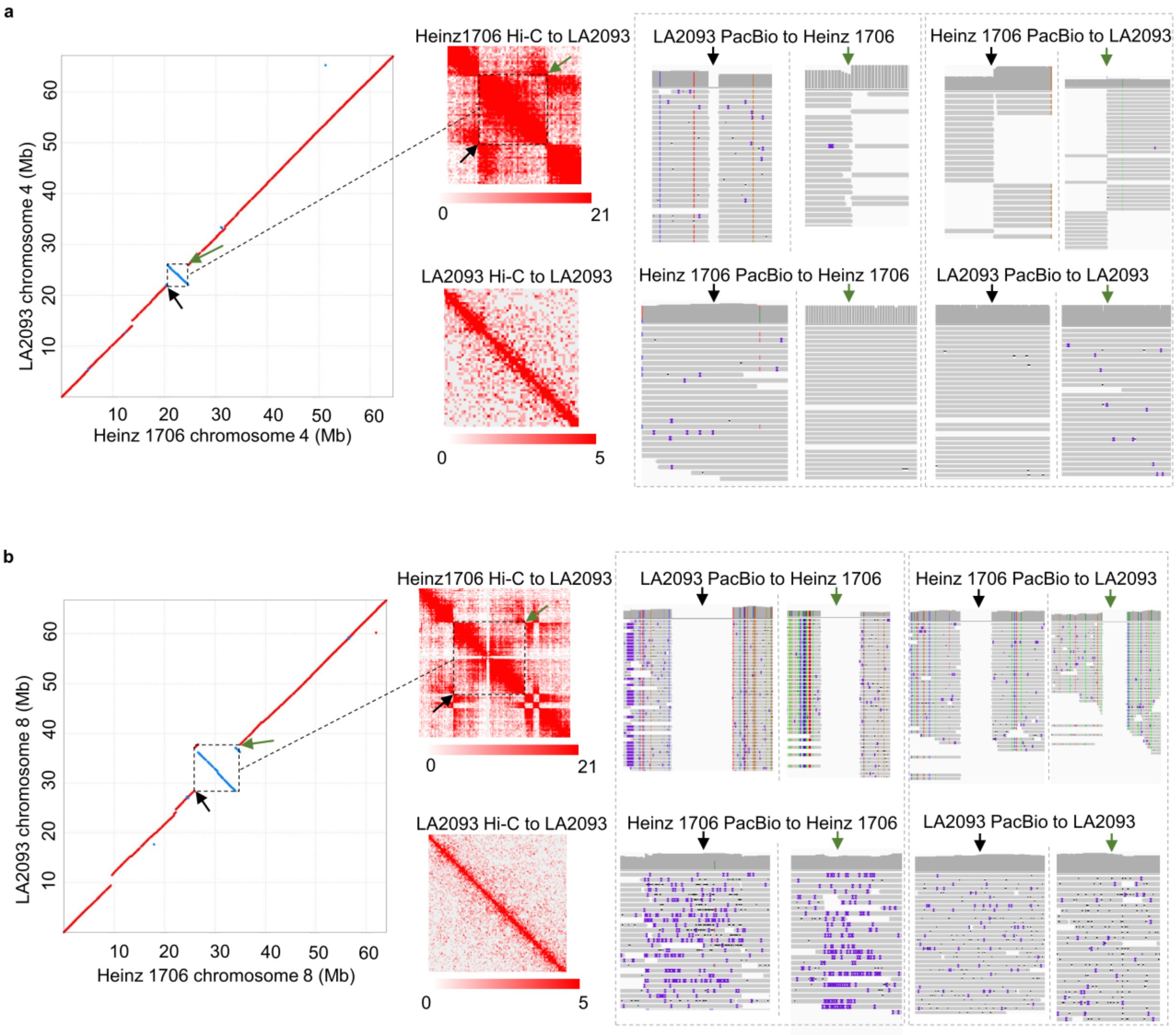

Supplementary Figure 5. Examples of inversions between LA2093 and Heinz 1706 supported by Hi-C contact maps and split PacBio read alignment. (**a**) chromosome 4. (**b**) chromosome 8. For each inversion, the two breakpoints were pointed by arrows with two different colors, black and green, respectively.

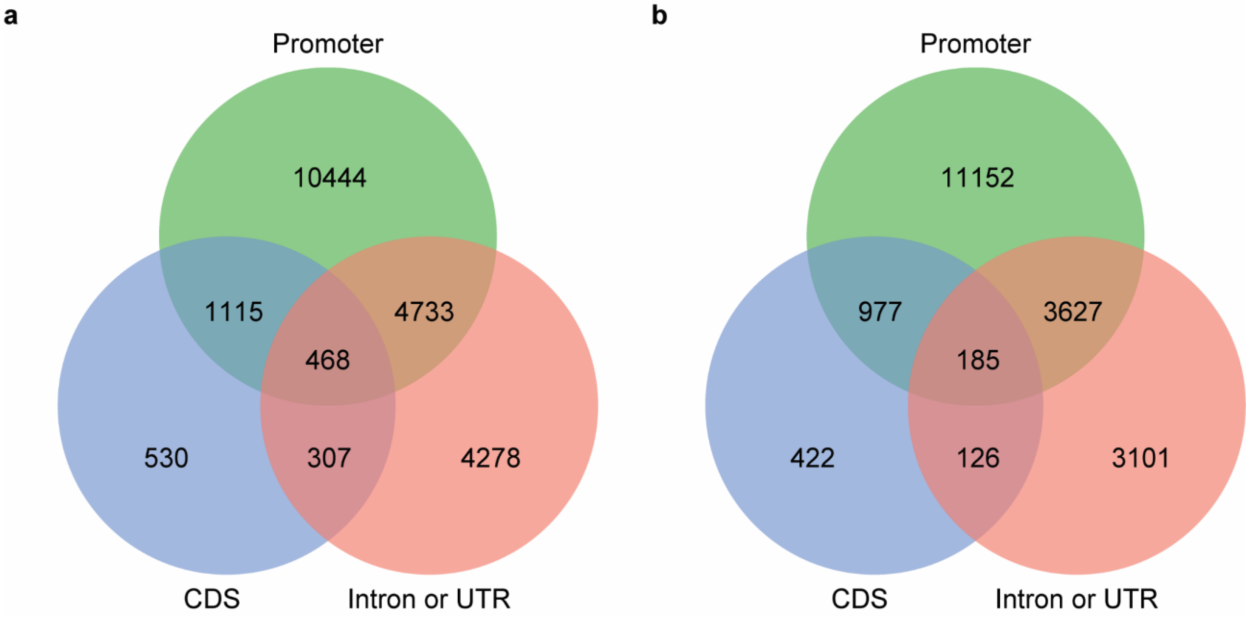

Supplementary Figure 6. Venn diagrams showing numbers of genes in LA2093 (a) and Heinz 1706 (b) having at SVs in the CDS, intron/UTR and promoter regions.

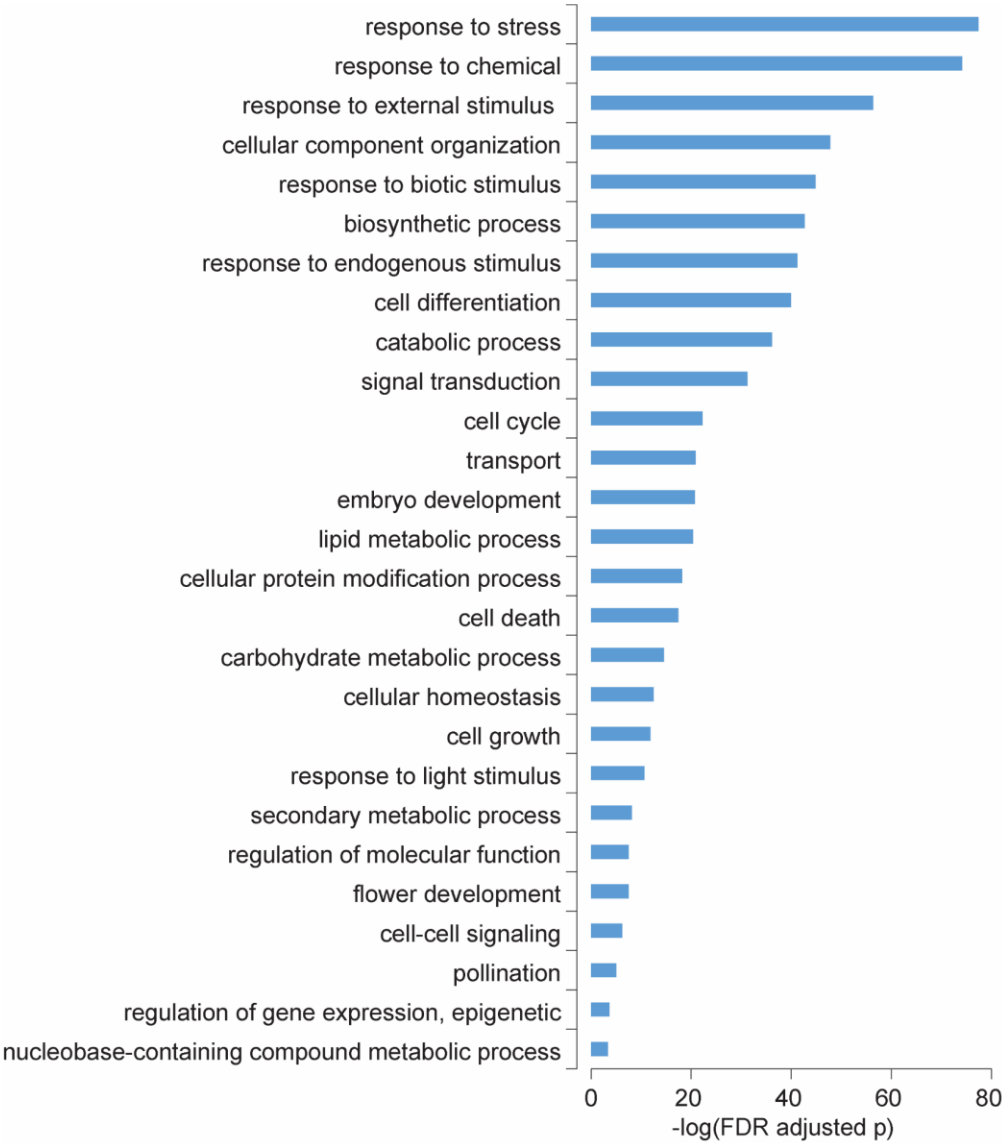

Supplementary Figure 7. GO terms enriched in genes with SVs in the gene body or promoter regions. Enriched GO terms were identified using two-tailed Fisher's exact test, adjusted for multiple comparisons.

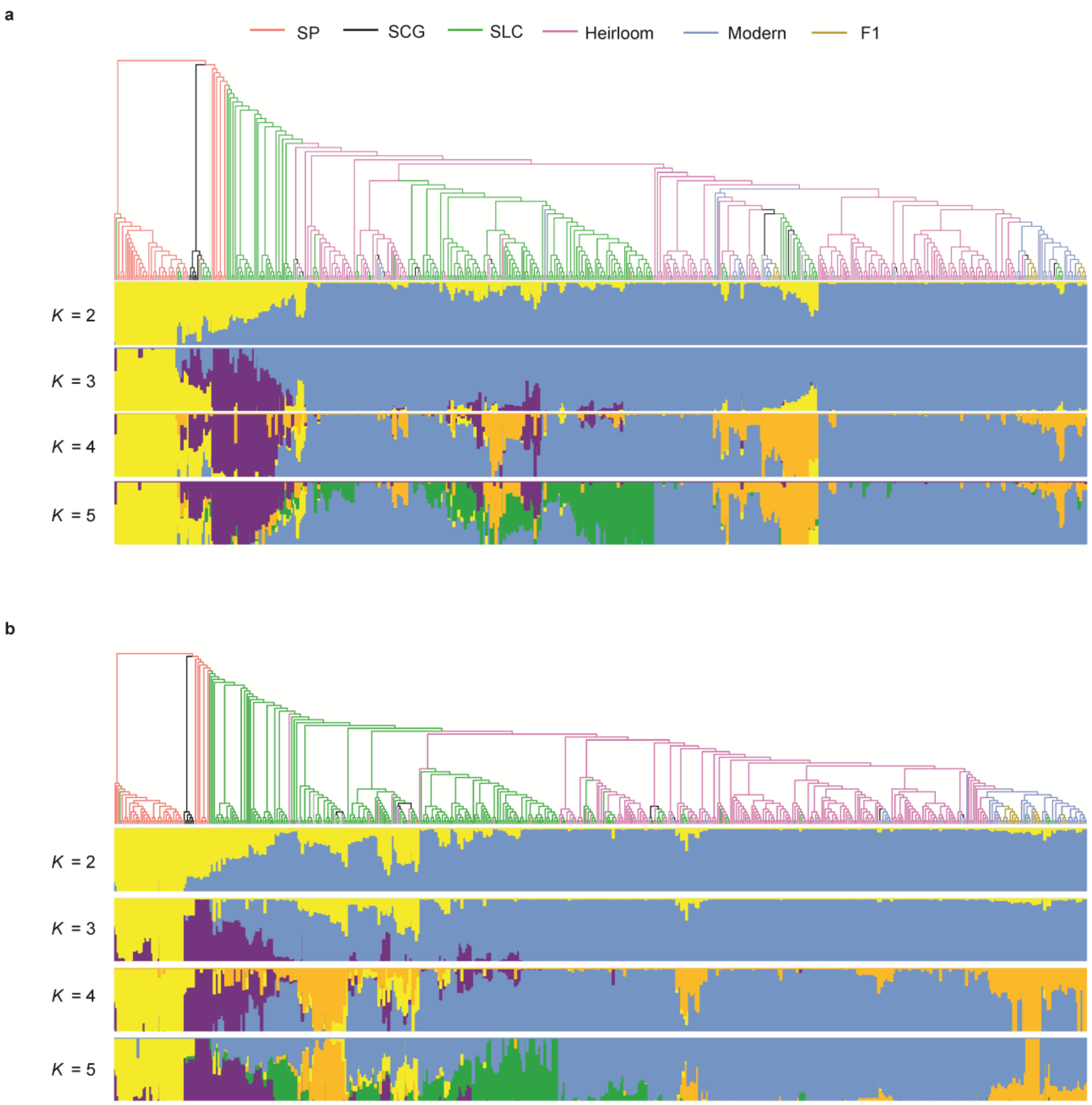

Supplementary Figure 8. Phylogenetic relationship and population structure of tomato accessions inferred using SVs (a) and SNPs (b). *K*, number of subpopulations.

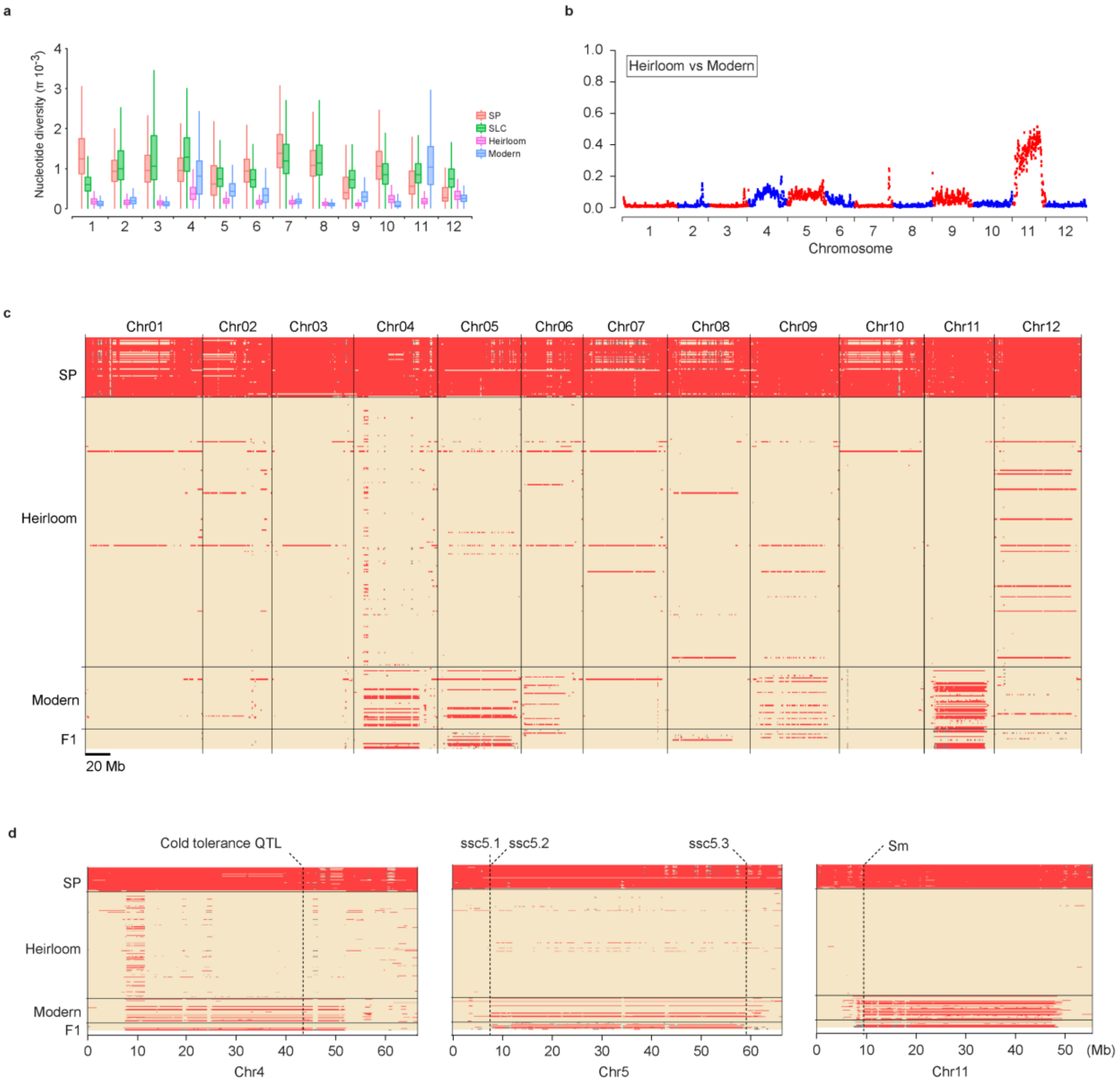

Supplementary Figure 9. Genome-wide analysis of introgressions in SLL heirloom, modern and F1 groups. a, Nucleotide diversity in different groups across the 12 chromosomes. For each box plot, the lower and upper bounds of the box indicate the first and third quartiles, respectively, and the center line indicates the median. The whisker represents 1.5× interquartile range of the lower or upper quartile. **b**, Distribution of F_{ST} values between heirloom and modern groups across the LA2093 genome. **c**, Introgressions from SP to modern and F1 tomatoes across the 12 tomato chromosomes. The introgression on chromosome 9 is known to be from *Solanum peruvianum* (Lin et al., 2014), which shares many common alleles with *S. pimpinellifolium*. **d**, Introgressions from SP to modern and F1 tomatoes on chromosomes 4, 5 and 11. Known QTLs and genes are indicated by the dash lines. Species-specific genome regions are shown in different colors, with SP in red and SLL in light yellow. Source data underlying Supplementary Figure 9a are provided as a Source Data file.

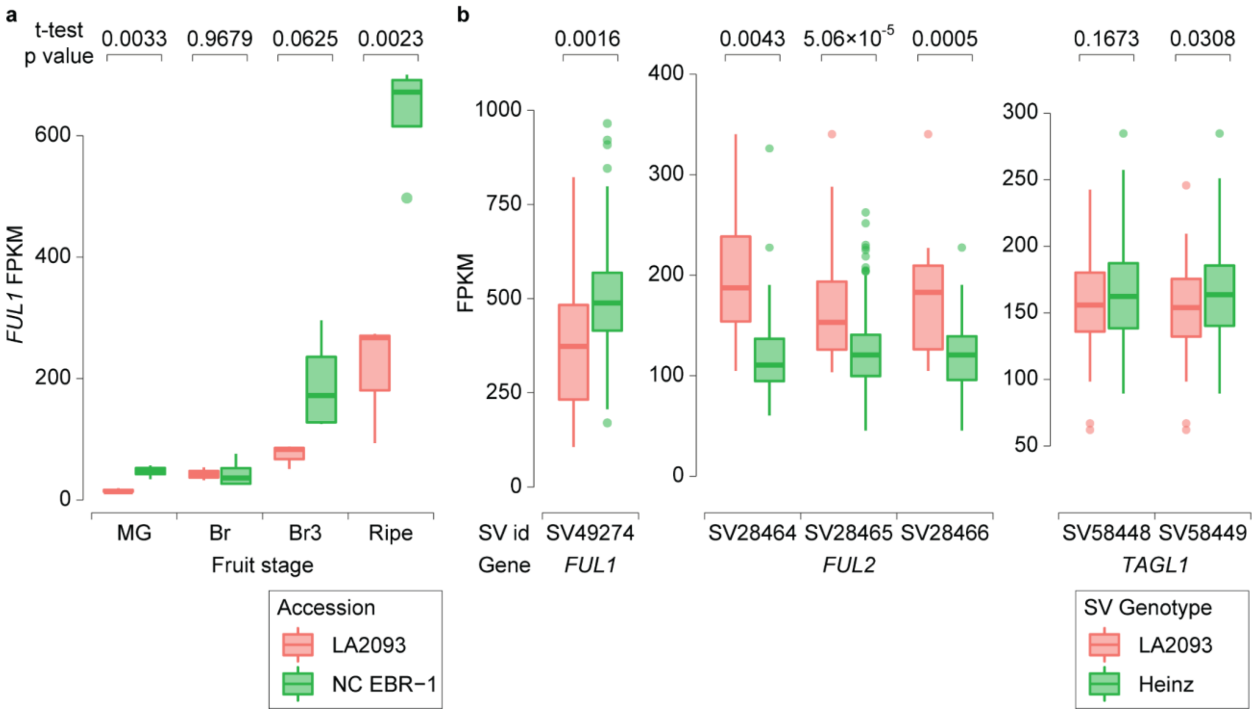

Supplementary Figure 10. Expression levels of *FUL1*, *FUL2* and *TAGL1* associated with the two alleles of the selected SVs. a, Expression of *FUL1* in LA2093 and NC EBR-1 during fruit development. NC EBR-1 carried the Heinz 1706 allele of SV49274. Expression was derived from the RNA-Seq data reported in Gao et al., 2019 with three or four biological replicates. Genotype of SV49274 in NC EBR-1 *FUL1* CDS was inferred based on the RNA-Seq data. Two-tailed Student's t-test was performed to compare *FUL1* expression levels between NC EBR-1 and LA2093 at each fruit developmental stage. **b**, Expression levels of *FUL1*, *FUL2* and *TAGL1* in tomato accessions carrying the homozygous LA2093 and Heinz 1706 alleles, respectively, of the selected SVs. For each SV from left to right, the numbers of accessions with homozygous LA2093 alleles are 17, 4, 13, 11, 56 and 58, and those with homozygous Heinz alleles are 238, 47, 189, 37, 175 and 191, respectively. Two-tailed Student's t-test was performed to compare expression levels of each gene between the accessions with homozygous LA2093 and with Heinz alleles for each SV. For each box plot, the lower and upper bounds of the box indicate the first and third quartiles, respectively, and the center line indicates the median. The whisker represents 1.5× interquartile range of the lower or upper quartile. Source data are provided as a Source Data file.

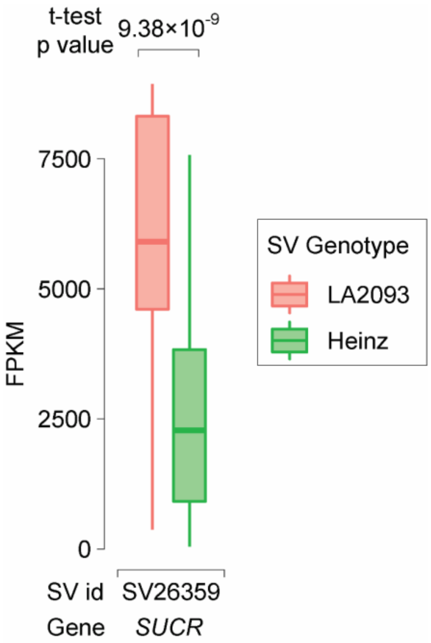

Supplementary Figure 11. Expression levels of *SUCR* in tomato accessions carrying the homozygous LA2093 and Heinz 1706 alleles, respectively, of the selected SV in the first intron of *SUCR*. The number of accessions with homozygous LA2093 and Heinz alleles are 11 and 186, respectively. Two-tailed Student's t-test was performed to compare expression levels of *SUCR* between the accessions with homozygous LA2093 and with Heinz alleles of SV26359. For each box plot, the lower and upper bounds of the box indicate the first and third quartiles, respectively, and the center line indicates the median. The whisker represents 1.5× interquartile range of the lower or upper quartile. Source data are provided as a Source Data file.

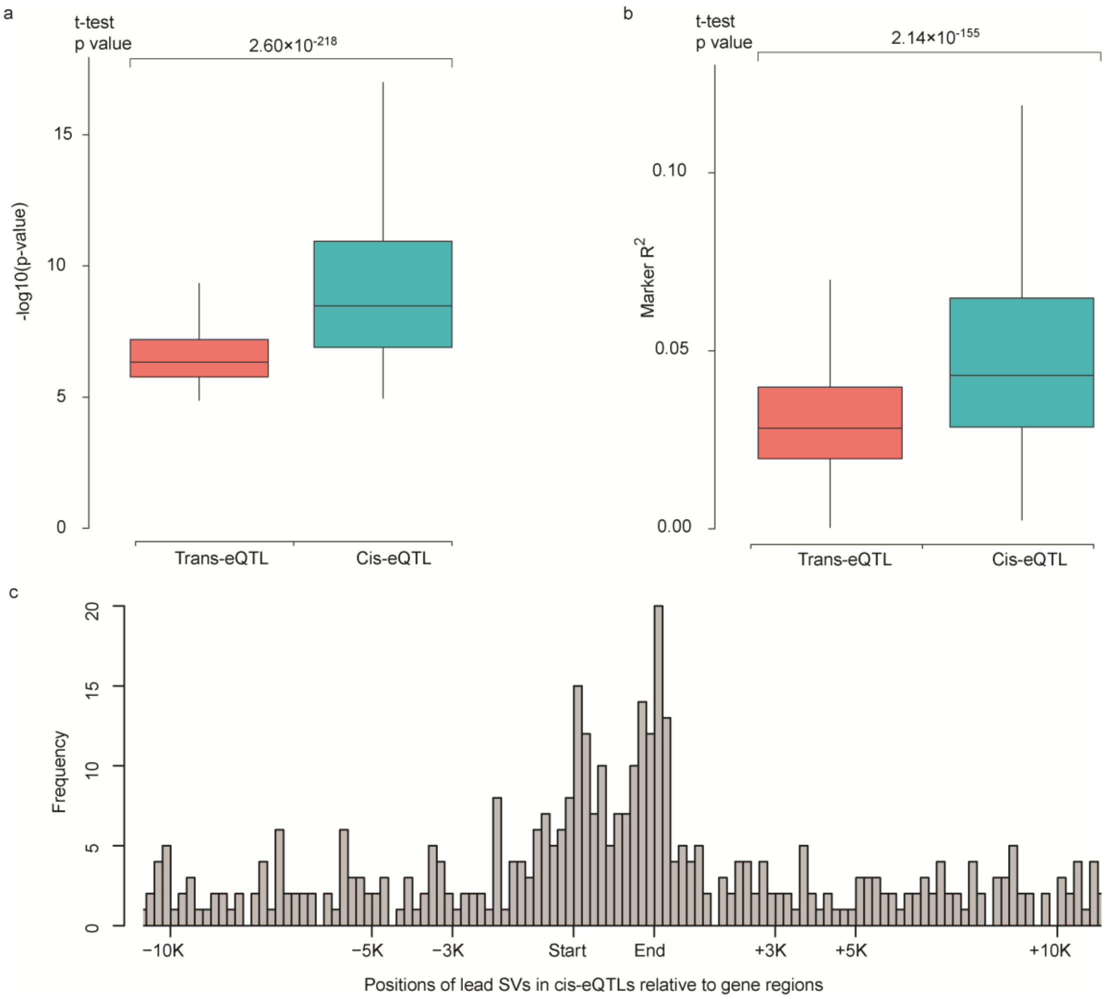

Supplementary Figure 12. Effects of cis- and trans-eQTLs on gene expression. a, Significance levels of cis- and trans-eQTLs. The numbers of cis- and trans-eQTLs are 2,708 and 8,081, respectively. Two-tailed Student's t-test was performed to compare significance levels between cis- and trans-eQTLs. **b,** Expression variation explained by cis- and trans-eQTLs. The numbers of marker R^2 values for cis- and trans-eQTLs are 2,728 and 8,313, respectively. Two-tailed Student's t-test was performed to compare the explained expression variation between cis- and trans-eQTLs. **c**, Distribution of cis-eQTLs surrounding gene regions. Each eQTL was represented by the lead SV. 'Start' and 'End' refer to the translational start and end sites. For each box plot in (**a**) and (**b**), the lower and upper bounds of the box indicate the first and third quartiles, respectively, and the center line indicates the median. The whisker represents 1.5× interquartile range of the lower or upper quartile. Source data underlying Supplementary Figure 12a and 12b are provided as a Source Data file.

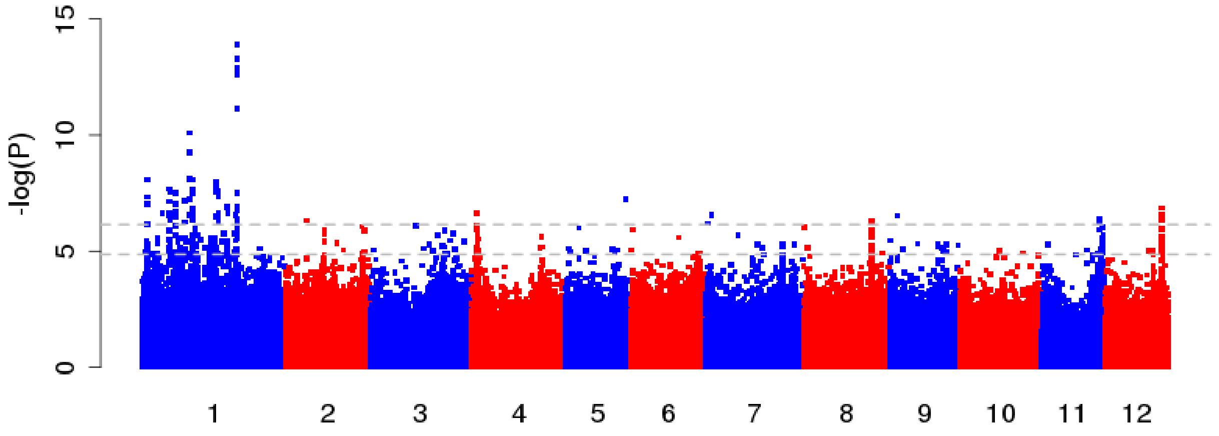

Supplementary Figure 13. Combined Manhattan plot of eQTLs associated with the expression of 17 flavonoid biosynthetic genes. The horizontal dashed lines correspond to the Bonferroni-corrected significance thresholds at α = 0.05 and α = 1.

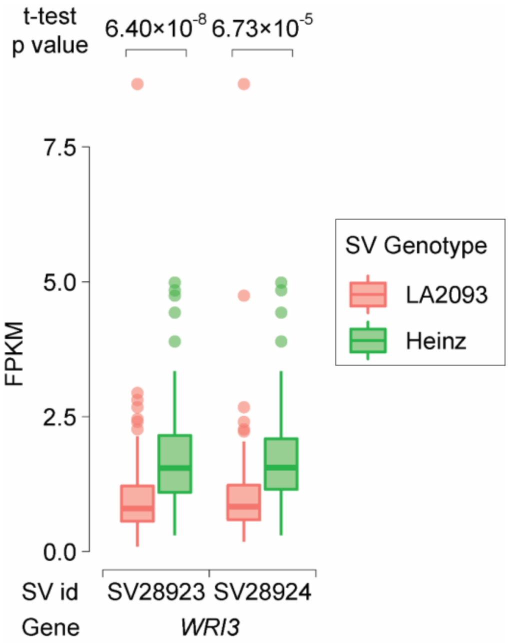

Supplementary Figure 14. Expression of *WRI3* in tomato accessions carrying the homozygous LA2093 and Heinz 1706 alleles, respectively, of SVs in the eQTL significantly associated with the *WRI3* expression. For each SV from left to right, the numbers of accessions with homozygous LA2093 alleles are 124 and 90, and those with homozygous Heinz alleles are 116 and 81, respectively. Two-tailed Student's t-test was performed to compare expression levels of *WRI3* between the accessions with homozygous LA2093 and with Heinz alleles for each SV. For each box plot, the lower and upper bounds of the box indicate the first and third quartiles, respectively, and the center line indicates the median. The whisker represents 1.5× interquartile range of the lower or upper quartile. Source data are provided as a Source Data file.

Supplementary Table 1. Quality evaluation of genome assemblies using Merqury (https://github.com/marbl/merqury).

Assembly	k-mers uniquely found only in the assembly	k-mers found in both assembly and the read set	QV	Error rate	solid k-mers in the assembly	solid k-mers in reads	Completeness (%)
LA2093	364,296	807,604,503	46	2.65E-05	402,237,383	404,142,869	99.5285
BGV006775	19,804,020	794,519,657	28	0.001484	395,551,843	404,231,551	97.8528

Supplementary Table 2. Summary statistics of repeat sequences in the LA2093 genome.

Class	Sub class	Count	Bases	% of genome
DNA transposon	DNA	9,070	2,499,631	0.31
	CMC-EnSpm	26,081	17,069,722	2.11
	MULE-MuDR	24,732	8,403,515	1.04
	PIF-Harbinger	15,500	3,415,549	0.42
	TcMar	437	50,333	0.01
	TcMar-Stowaway	4,314	1,078,695	0.13
	hAT-Ac	11,193	4,118,038	0.51
	hAT-Charlie	231	68,363	0.01
	hAT-Tag1	756	284,906	0.04
	hAT-Tip100	14,530	2,700,353	0.33
LINE	L1	14,527	11,103,971	1.37
	Penelope	96	14,267	0.00
	RTE-BovB	19,574	4,251,431	0.53
Retrotransposon	LTR	6,581	2,429,991	0.30
	Caulimovirus	6,007	8,030,234	0.99
	Copia	82,138	58,919,526	7.29
	ERV1	1,144	385,093	0.05
	Gypsy	322,569	305,709,718	37.84
	Pao	1,022	6,006,707	0.74
RC	Helitron	2,358	1,034,074	0.13
Others	MITE	138,774	58,179,754	7.20
	Retroposon	7,836	1,874,365	0.23
	SINE	8,106	1,555,633	0.19
	tRNA-RTE	851	95,638	0.01
	Satellite	162	160,985	0.02
	Simple repeat	125	47,102	0.01
	Unknown	185,331	44,753,849	5.54
Total		904,216	544,325,865	67.38

Supplementary Table 3. Distribution of indels in different genomic regions.

Region	LA2093		Heinz 1706	
	**Genome**	**Indel region**	**Genome**	**Indel region**
Total length (bp)	807,856,003 (100.0%)	46,924,267 (100.0%)	782,520,033 (100.0%)	32,748,340 (100.0%)
Gene body (bp)	164,953,612 (20.4%)	4,660,265 (9.9%)	122,213,808 (15.6%)	1,734,158 (5.3%)
CDS (bp)	37,964,886 (4.7%)	645,474 (1.4%)	34,730,951 (4.4%)	381,763 (1.2%)
Promoter (bp)	75,421,083 (9.3%)	2,967,214 (6.3%)	77,467,574 (9.9%)	2,460,860 (7.5%)

Supplementary Table 4. Summary statistics of SV genotyping using Illumina short reads in the reference accessions.

	Heinz			LA2093		
	Number	% of total	% of (total-undetermined)	Number	% of total	% of (total-undetermined)
Homozygous Heinz genotype	75,132	78.61	98.01	1,315	1.38	1.77
Homozygous LA2093 genotype	1,171	1.23	1.53	72,798	76.17	97.85
Heterozygous	355	0.37	0.46	282	0.3	0.38
Undetermined	18,913	19.79	-	21,176	22.16	-
Total	95,571	-	-	95,571	-	-

Supplementary Table 5. Evaluation of the SV imputation accuracy.

Accession	Group	Proportion of data masked as missing	Missing data filled rate	Imputation accuracy
BGV006865	*S. lycopersicum var cerasiforme*	10%	93.14%	98.33%
BGV006865	*S. lycopersicum var cerasiforme*	20%	93.29%	98.52%
BGV006865	*S. lycopersicum var cerasiforme*	30%	93.29%	98.35%
BGV007181	*S. pimpinellifolium*	10%	92.26%	97.82%
BGV007181	*S. pimpinellifolium*	20%	92.22%	98.09%
BGV007181	*S. pimpinellifolium*	30%	91.54%	98.22%
BGV007865	*S. lycopersicum L.*	10%	97.96%	99.55%
BGV007865	*S. lycopersicum L.*	20%	97.66%	99.52%
BGV007865	*S. lycopersicum L.*	30%	97.72%	99.51%
