# Supplementary material for: Genome of Solanum pimpinellifolium provides insights into structural variants during tomato breeding
Source: Nat Commun. 2020 Nov 16;11:5817. doi: 10.1038/s41467-020-19682-0 (PMC7670462; doi:10.1038/s41467-020-19682-0)
Supplement: Supplementary file 3 — Reporting Summary [file 41467_2020_19682_MOESM3_ESM.pdf]

nature research

Corresponding author(s): Zhangjun Fei & Shan Wu

Last updated by author(s): Oct 17, 2020

Reporting Summary

Nature Research wishes to improve the reproducibility of the work that we publish. This form provides structure for consistency and transparency in reporting. For further information on Nature Research policies, see our Editorial Policies and the Editorial Policy Checklist.

Statistics

For all statistical analyses, confirm that the following items are present in the figure legend, table legend, main text, or Methods section.

n/a	Confirmed	
☐	☒	The exact sample size (n) for each experimental group/condition, given as a discrete number and unit of measurement
☐	☒	A statement on whether measurements were taken from distinct samples or whether the same sample was measured repeatedly
☐	☒	The statistical test(s) used AND whether they are one- or two-sided<br>*Only common tests should be described solely by name; describe more complex techniques in the Methods section.*
☒	☐	A description of all covariates tested
☐	☒	A description of any assumptions or corrections, such as tests of normality and adjustment for multiple comparisons
☐	☒	A full description of the statistical parameters including central tendency (e.g. means) or other basic estimates (e.g. regression coefficient) AND variation (e.g. standard deviation) or associated estimates of uncertainty (e.g. confidence intervals)
☐	☒	For null hypothesis testing, the test statistic (e.g. F, t, r) with confidence intervals, effect sizes, degrees of freedom and P value noted<br>*Give P values as exact values whenever suitable.*
☒	☐	For Bayesian analysis, information on the choice of priors and Markov chain Monte Carlo settings
☒	☐	For hierarchical and complex designs, identification of the appropriate level for tests and full reporting of outcomes
☐	☒	Estimates of effect sizes (e.g. Cohen's d, Pearson's r), indicating how they were calculated

Our web collection on statistics for biologists contains articles on many of the points above.

Software and code

Policy information about availability of computer code

Data collection	No software was used in data collection.
Data analysis	We used freely available bioinformatics softwares, including:Guppy(v3.1.5), CANU(v1.7.1), Trimmomatic(v0.36), BWA-MEM(v0.7.17), Pilon (v1.22), redundans(v0.14a), SALSA(v2.2), RaGOO(v1.1), ALLMAPS(v0.8.12), MITE-Hunter(v11-2011), LTRharvest(v1.5.10), RepeatMasker (v4.0.8), RepeatModeler(v1.0.11), HISAT2(v2.1), StringTie(v1.3.3b), PASA(v2.3.3), BRAKER(v2.0.4), GeneMark-ET(v4.61), SNAP(v2006-07-28), Maker(v2.31.10), Minimap2(v2.17), Assemblytics(v1.1), pbsv(v2.2), SVIM(v1.2.0), Blast2GO(v1.3.11), MUMmer4(v4.0.0), NECAT(v0.0.1), fmlrc (v0.1.2), Picard(v2.3), GATK(v3.8), IQ-TREE(v1.6.8), VCFtools(v0.1.16), EMMAX(v20120210), fillGenotype, PopLDdecay(v3.27), and hot_scan. Custom computational pipelines related to SV detection and genotyping can be accessed through https://github.com/GaoLei-bio/SV.

For manuscripts utilizing custom algorithms or software that are central to the research but not yet described in published literature, software must be made available to editors and reviewers. We strongly encourage code deposition in a community repository (e.g. GitHub). See the Nature Research guidelines for submitting code & software for further information.

Data

Policy information about availability of data

All manuscripts must include a data availability statement. This statement should provide the following information, where applicable:

- Accession codes, unique identifiers, or web links for publicly available datasets
- A list of figures that have associated raw data
- A description of any restrictions on data availability

The genome sequence of Solanum pimpinellifolium LA2093 has been deposited at DDBJ/ENA/GenBank under the accession JAAONP000000000. The version described in this paper is JAAONP010000000. Raw genome and transcriptome sequencing reads have been deposited into the NCBI BioProject database under accession PRJNA607731. The genome sequence of LA2093 and the associated annotations are also available at Sol Genomics Network (https://solgenomics.net/).

April 2020

The SV genotyping data in the 597 tomato accessions is available via Figshare (https://figshare.com/articles/dataset/tomato_SV_matrix/12922151).

Field-specific reporting

Please select the one below that is the best fit for your research. If you are not sure, read the appropriate sections before making your selection.

[x] Life sciences [ ] Behavioural & social sciences [ ] Ecological, evolutionary & environmental sciences

For a reference copy of the document with all sections, see nature.com/documents/nr-reporting-summary-flat.pdf

Life sciences study design

All studies must disclose on these points even when the disclosure is negative.

Sample size	For SV and SNP genotyping in the tomato population, 725 public available NCBI-SRA samples were used. For tomato orange-stage fruit transcriptome analysis, 399 public available NCBI-SRA samples were used. Sample sizes for other experiments were chosen based on the testable hypotheses. For LA2093 fruit RNA-Seq experiment, three biological replicates were used.
Data exclusions	Data exclusion criteria were pre-established as described in the Methods. For example, 128 out of 725 accessions with less than 40% of SVs genotyped were excluded from downstream analyses.
Replication	The LA2093 fruit RNA-Seq experiment were successfully replicated three times with different plants.
Randomization	Samples are classified into different species and groups based on NCBI metadata.
Blinding	Samples were labeled with IDs that did not indicate their taxonomic information. Thus the samples were not selected based on expected results.

Reporting for specific materials, systems and methods

We require information from authors about some types of materials, experimental systems and methods used in many studies. Here, indicate whether each material, system or method listed is relevant to your study. If you are not sure if a list item applies to your research, read the appropriate section before selecting a response.

Materials & experimental systems

n/a	Involved in the study
[x]	[ ] Antibodies
[x]	[ ] Eukaryotic cell lines
[x]	[ ] Palaeontology and archaeology
[x]	[ ] Animals and other organisms
[x]	[ ] Human research participants
[x]	[ ] Clinical data
[x]	[ ] Dual use research of concern

Methods

n/a	Involved in the study
[x]	[ ] ChIP-seq
[x]	[ ] Flow cytometry
[x]	[ ] MRI-based neuroimaging
