# Supplementary material for: Genome of Solanum pimpinellifolium provides insights into structural variants during tomato breeding
Source: Nat Commun. 2020 Nov 16;11:5817. doi: 10.1038/s41467-020-19682-0 (PMC7670462; doi:10.1038/s41467-020-19682-0)
Supplement: Supplementary file 4 — Description of Additional Supplementary Files [file 41467_2020_19682_MOESM4_ESM.pdf]

Description of Additional Supplementary Files

Supplementary Data 1
Summary statistics of the tomato genome assemblies.

Supplementary Data 2
Inversions between genomes of LA2093 and ‘Heinz 1706’ and allele frequency dynamics in different tomato populations

Supplementary Data 3
Genes located in the inversion regions between the LA2093 and 'Heinz 1706' genomes

Supplementary Data 4
Structural variations (SVs) detected between LA2093 and ‘Heinz 1706’ and allele frequency dynamics in different tomato populations

Supplementary Data 5
Selected SVs in genes known to control horticultural traits

Supplementary Data 6
List of tomato accessions used for tomato SV genotyping

Supplementary Data 7
List of identified eQTLs

Supplementary Data 8
List of trans-acting eQTL hotspots

Supplementary Data 9
List of genes targeted by the MYB12-containing eQTL hotspot on chromosome 1

Supplementary Data 10
eQTLs associated with the expression of flavonoid biosynthetic genes

Supplementary Data 11
List of genes targeted by the WRI3-containing eQTL hotspot on chromosome 3
