# Supplementary material for: Genome of Solanum pimpinellifolium provides insights into structural variants during tomato breeding
Source: Nat Commun. 2020 Nov 16;11:5817. doi: 10.1038/s41467-020-19682-0 (PMC7670462; doi:10.1038/s41467-020-19682-0)
Supplement: Supplementary file 2 — Peer Review [file 41467_2020_19682_MOESM2_ESM.pdf]

Reviewer #1 (Remarks to the Author):

The MS describes the high quality genome sequence of the wild species Solanum pimpinellifolium (cv LA2093). It compares the sequence to the one of the cultivated tomato and identified 28 inversions and several structural variants (SV). Those SV were genotyped in a large set of accessions and used to map QTL for fruit traits and eQTL, identifying new candidate genes. The MS identified the role of many SV in the variation of all the traits and provides a large set of data important for the whole tomato community. The MS represents an original study underlying the role of SV in tomato evolution. It is clear and well written and the methods used are relevant.

It could be interesting to compare the power of SV and SNP in GWAS study. Finally in order to have a broader impact the authors could provide as supplemental (or on a dedicated website) a list of all the SV genotypes in the panel studied as well as a table of correspondence of the genes annotated in S. pimpinellifolium and S. lycopersicum. I thus recommand to accept it for publication

Minor revisions :
- On chromosome 11, the introgression detected is supposed to be related to the presence of the Sm resistance gene; I wonder if it is present in all the F1 showing introgression traces or if it could not be related to the presence of other disease resistance genes on this chromosome, like I-2 (against fusarium, much more frequent) or Ty-2 (against TYLCV).
- 3 lines before Discussion : Protomer instead of Promoter
Mathilde Causse

Reviewer #2 (Remarks to the Author):

The authors of the manuscript “Genome of Solanum pimpinellifolium provides insights into structural variants during tomato breeding” describe an interesting study that highlights the role of SV and smaller indels in the evolution of SP. They constructed a reference quality assembly for LA2093 and compared this to the well used Heinz accession. Furthermore, they utilized the genotyping approach of the so identified SV to learn more about their potential impact on RNA expression and thus phenotypes across the cultivar. Overall the manuscript is well written and easy to follow. In the following I list my questions and concerns in no particular order:

1. I noticed you detect the SV based on a genomic alignment. How do you handle the heterozygous SV or the detection of their genotypes? This can become a problem as given the assembly alone you will miss multiple SV and you seem to rely on the assembly.
2. I am maybe a bit confused as you are also using a mapping approach but I am not clear how they were put together, but I think you used the mapping to only confirm the SV detected by assembly??
3. I noticed that you described the detection of insertions, deletions and inversions, but no duplications or translocations were detected. Was that by design? And if so why did you ignore these?
4. I would suggest to redefine indel vs. SV sizes. Typically indels (2bp -50bp) are much smaller than SV (50bp+). That would make it later in the text easier to distinguish.
5. Did you observed the standard length distributions across the indels?
6. It is surprising that 21,875 and 19,590 of the predicted genes were impacted by at least one indel. Is that due to the assembly strategy or the lack of improving the accuracy? I somehow doubt that it is a biological signal as this would be very disruptive. Are these indels in introns vs. exons ?
7. I noticed that you implemented your own SV genotyping approach and confirmed that using ONT for one sample. I would encourage you also to report how many SV you found in addition to the genotyped SV for that comparison. Also I might have overlooked it but I don’t see a description in the methods how the ONT reads were processed and SV were called.
8. Maybe I missed it but when you are talking about FUL1+2 can you maybe describe the deletions

and insertions more. Are they frameshifts, are they in their exons? Like you do it for SUCR. Furthermore, I noticed that for some of these listed events for certain genes you list the population frequency and for some not. Would be interesting to make that more consistent as these are important events.
9. I noticed you have specified the versions of some tools (e.g. Canu) but left that out for many. Please add this as this improves reproducibility of your study.
10. Are the scripts etc. available? I didn't see these referenced in the main text or methods. As you seem to have implement a lot of your own procedures/methods yourself (e.g. SV genotyping) are these programs available and benchmarked ? Please make these public available etc.

Reviewer #3 (Remarks to the Author):

Tomato is an important model for studying how eukaryotic genome structure influences genome function. The manuscript by Wang and Gao et al. is a major addition to this field, and will be of broad interest to the research community. Overall I find the work to be thoughtful, valid, and methodologically sound. I also find the conclusions to be reasonable given the data and analysis available. The paper presents a high-quality and curated S. pimpinellifolium reference genome as an important resource for the Solanaceae community. I believe this assembly and associated annotations will have much utility for a large number of researchers/projects.

However, some key findings are not novel/original because they were first described in the recently published paper (Alonge and Wang et al. Cell, 2020), a reference to which is missing from the manuscript. However, some findings are still novel and of great interest, including the finding of many candidate mutations associated with known genes underlying important traits. Additionally, the eQTL analysis identifying putative regulatory hubs presents major findings. Though the manuscript gives promising results regarding variants associated with important genes, the manuscript lacks functional genetics analysis to go beyond statistical association and directly assess the functional impact of candidate QTL.

Altogether, I commend the authors for their work. Please see below for more detailed comments as well as additional minor points that I believe would improve the clarity and impact of the manuscript.

Major Comments

Comment 1:
The manuscript omits some necessary references and/or descriptions of previous work. Firstly, the authors state that "draft genome assemblies of two accessions, LA1589 (ref. 4) and LA0480 (ref. 5), have been generated using Illumina short-read sequencing technology. Although these assemblies provided some essential genomic information for SP, they are highly fragmented and incomplete,". However, a chromosome-scale long-read SP assembly (accession BGV006775) was published in Alonge et. al. 2019 (ref. 52). This should be included in the description of previous SP genome assemblies. This assembly is likely of lower base-accuracy and did not undergo de novo scaffolding, so the new LA2093 assembly is probably superior. Still, the previous assembly should be mentioned and cited in the introduction, and some comparisons should be made.

Secondly, Alonge and Wang et al., Cell, 2020 was recently published that is highly related to this manuscript but is not referenced. This publication must be cited and described in the introduction and in parts of the results section. For example, the sentence "However, the distribution of SVs in tomato accessions and their population dynamics are largely unexplored." is no longer true. Also, Alonge and Wang et al. Cell, 2020 describes sb1, another "suppressor of branching" QTL, and this should be mentioned along with sb3 in the introduction of known SVs influencing traits.

The Alonge and Wang et al 2020 publication renders some of Wang and Gao et al. as not novel. Please see comment 2 for more details. However, much of the work is still novel and has not been done before in tomato, such as the inversion analysis or the eQTL analysis. Furthermore, Alonge and Wang et al 2020 only examined 100 accessions with long-reads and did not genotype SVs in a larger set of accessions with short-reads. Wang and Gao et al. also provide valuable results about new potential variants underlying important traits, though functional validation is lacking (see comment 7)

Comment 2:
I believe the introgression analysis (Figure 2 and S9) is not original/novel since these introgressions and others were described in great detail in Alonge and Wang et al 2020. At a minimum the authors should refer to these results as being previously described by Alonge and Wang et al 2020.

Comment 3:
Using Hi-C data, the authors show compelling evidence that the LA2093 inversions are real. However, I have a few comments regarding the analysis of diversity and recombination in the inverted regions. Firstly, what is meant by "diversity" in the results text and especially in Figure S6. This should be defined in the methods and caption, respectively. Secondly, their rationale for reduced recombination in the chr1 inversion region is unclear to me. The authors state that "restriction of recombination was detected around the tomato fruit weight QTL, fw1.1". However, LA2093 was not used as a parent in the fw1.1 mapping populations. Furthermore, the referenced fw1.1 paper states that 2/3 of chromosome 1 experienced reduced recombination, while the inversion described in this manuscript is only 14 Mbp long. The authors state in the introduction that LA2093 is a parent in a widely used RIL population. Therefore, can they use this data from this RIL population to assess recombination rates in the inverted regions? Broadly, if the authors intend to discuss reduced recombination rates, the conclusions should be much more clear.

Comment 4:
The authors forgo published short-read SV genotyping tools such as Paragraph (Chen et al, Genome Biology, 2019) and vg (Hickey et al, Genome Biology, 2020) and instead use a custom method. The authors provide evidence that the pipeline yields high specificity with their comparison of LA1589 long-read based SV calls. However, their analysis does not measure the sensitivity of their SV genotyping method. The authors state that 96.7% of the LA1589 genotyped SVs were confirmed with long-read LA1589 SV calling. But of the long-read SVs shared between LA1589 and LA2093, what percentage of them were genotyped with short-read data? The authors should also consider genotyping LA2093 SVs with LA2093 short reads to measure sensitivity/specificity. This would provide sensitivity information in addition to the specificity measurements given in the manuscript. This is necessary because the authors are using an unpublished method to genotype SVs with short reads.

Comment 5:
I commend the authors for a professionally established reference genome. The authors properly screen for contamination and false duplications. They also provide gene and repeat annotations. The authors must also provide a QV estimate with a tool such as mercury (https://github.com/marbl/merqury). I also encourage the authors to measure how many "pan-genome genes" (Gao et al. 2019) were annotated in LA2093. Finally, please ensure that each LA2093 gene has a mapping to its homolog in ITAG4.*. This should be encoded in the GFF file. The authors may consider generally comparing the LA2093 genes with ITAG4.*.

Comment 6:
Throughout the manuscript, the authors use two variant sets, one with respect to SL4.0 and one with respect to LA2093. For each analysis, it should be stated which assembly is being used as the reference. For example, exactly which reference/variants are used to build the trees in Figure S8, or for the eQTL analysis? Relatedly, this statement was unclear: "homozygous LA2093 alleles of the SVs were prevalent, taking up an average of 58.4% of the SVs in each accession, while only 39.4% SVs

were homozygous for the Heinz 1706 genotypes". This analysis should clarify how the variants are defined with respect to a reference genome. If using SL4.0 as the reference, homozygous 1706 alleles are not "variants". So are these percentages with respect to two different coordinates systems?

Comment 7:
The section "SV selection associated with breeding traits" presents very exciting results and underscores how much there is to discover when considering structural variation in genotype to phenotype studies. It is disappointing that no functional genetics was used to demonstrate causality for some of the putative variants underlying breeding traits. Still, the results are impactful. For most of the candidate variants, increasing (with domestication/modernization) allele frequency was paired with differential expression analysis to implicate candidate variants. Why was a differential expression analysis not performed for the Lin5 or AgpL1 examples? Also, what exactly is being shown in Figure S10A? Do the boxplots describe the distribution over biological replicates? It is confusing since this panel is only comparing two genotypes. And why is this the only expression analysis that uses RPKM rather than FPKM? This is not described in the methods -- all RNA-seq analysis and associated statistical (e.g. Figure S10, S11, and S14) methods should be described in the methods.

Minor Comments

Comment 8:
On multiple occasions, the authors use the word "significant" without providing the statistical rationale (such as a p-value) inline. Many of the p-values and statistical tests are described in the methods, but they should also be presented in parentheses after sentences in the results section.

Comment 9:
Figures 2G and S9 are not interpretable given the caption. The caption should give some description of how to interpret the figure and can refer to the methods for more details.

Comment 10:
"... SP has been used as an important germplasm donor in modern breeding of tomato". This should be changed to "... SP has been used as an important germplasm donor in modern tomato breeding".

Comment 11:
I recommend not using the term "high-confidence" when describing the variant calls. This is appropriate when there is a counterpart set (like "low confidence", e.g. wheat reference gene models).

Comment 12:
"Genotyping these SVs in ~600 representative tomato accessions unravels alleles" - The word "unravels" is not appropriate here.

Comment 13:
"discovers numerous novel SVs underlying genes known to regulate important breeding traits such as fruit weight and lycopene content" - this should be rephrased since an SV cannot "underly" a gene.

Comment 14:
The term "master regulator" is conventionally used for pioneer transcription factors. The authors should consider if their use of this term is appropriate and/or use an alternative description.

Comment 15:
Black arrows for figure S5 should indicate a "start" and "stop" (or similar). The authors align PacBio reads to confirm the inversions. I suggest also aligning LA2093 reads to the LA2093 assembly to show that alignments are not discordant at inversion breakpoints.

Comment 16:
"SVs in gene body and promoter regions can directly impact gene functions and expression" - This should be rephrased to consider both direct and indirect impacts.

Comment 17:
"Only 14.8% of identified indels overlapped with gene body and promoter" - rephrase as "gene body or promoter"

Comment 18:
Figure S8 should define what "k" is in the caption.

Comment 19:
The methods used to generate Figure S3 are not described

Comment 20:
"SVs were prevalent, taking up an average of 58.4% of the SVs in each accession" - This sentence is unclear (also see comment 6).

Comment 21:
Following conventions in the literature, "cis" and "trans" should be used instead of "local" and "distant" eQTL.

Comment 22:
"eQTLs were located near the start and end of coding regions" - rephrase as " start or end"

Comment 23:
"Hi-C data and synteny with the Heinz1706 genome were integrated to construct the pseudomolecules." - The authors should be more specific about this process. Exactly how was the Hi-C and synteny information combined? Also, please provide the assembly statistics for the initial SALSA scaffolds.

Comment 24:
The discussion is a narrow summary of the results. The authors should expand the discussion to describe the broad implications of this work. The authors should also emphasize the importance of tomato as a model, either in the discussion or the introduction, since many readers may not know that some of the leading work in this field is in tomato.

Reviewer #1

The MS describes the high quality genome sequence of the wild species *Solanum pimpinellifolium* (cv LA2093). It compares the sequence to the one of the cultivated tomato and identified 28 inversions and several structural variants (SV). Those SV were genotyped in a large set of accessions and used to map QTL for fruit traits and eQTL, identifying new candidate genes. The MS identified the role of many SV in the variation of all the traits and provides a large set of data important for the whole tomato community. The MS represents an original study underlying the role of SV in tomato evolution. It is clear and well written and the methods used are relevant.

It could be interesting to compare the power of SV and SNP in GWAS study.
Response: We thank the reviewer for the suggestion. We did use SNPs in our eQTL analysis and found the results are largely consistent with those obtained using SVs. Please check the following figure for an example. Since the focus of our manuscript is on SVs, we prefer not to include the SNP-eQTL results.

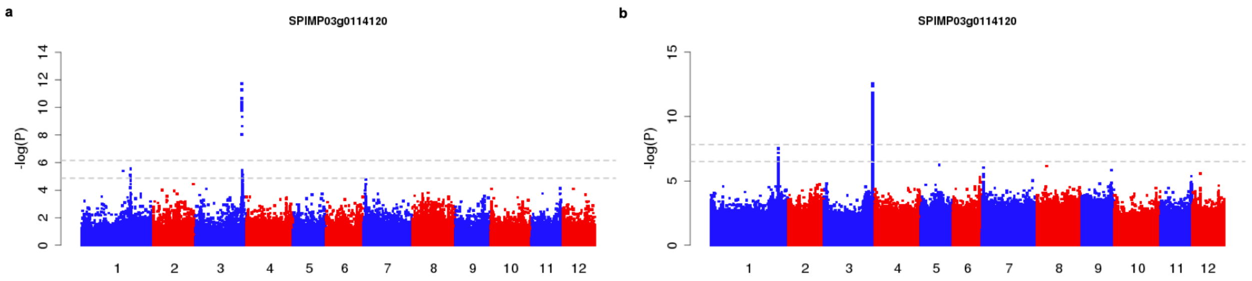

eQTL analysis of the *WRI3* gene (*SPIMP03g011412*) using SVs (a) or SNPs (b)

Finally in order to have a broader impact the authors could provide as supplemental (or on a dedicated website) a list of all the SV genotypes in the panel studied as well as a table of correspondence of the genes annotated in *S. pimpinellifolium* and *S. lycopersicum*. I thus recommend to accept it for publication
Response: We thank the reviewer for the suggestion. A file listing all the SV genotypes in the panel has been deposited in FigShare and the link has been added in the “Data Availability” section (Line 623-624).

Minor revisions:
- On chromosome 11, the introgression detected is supposed to be related to the presence of the Sm resistance gene; I wonder if it is present in all the F1 showing introgression traces or if it could not be related to the presence of other disease resistance genes on this chromosome, like I-2 (against fusarium, much more frequent) or Ty-2 (against TYLCV).
Response: We have added the introgression analysis result for the F1 accessions (Supplementary Fig. 9). Regarding the introgression on chromosome 11, around half of the F1 accessions harbor the introgression, and the introgression does not contain *I-2* and *Ty-2*. Regions containing *I-2* and *Ty-2* in modern and F1 tomato accessions, if present, are believed to be introgressed from wild species other than SP.

- 3 lines before Discussion: Protomer instead of Promoter
Response: Corrected.

Reviewer #2

The authors of the manuscript "Genome of *Solanum pimpinellifolium* provides insights into structural variants during tomato breeding" describe an interesting study that highlights the role of SV and smaller indels in the evolution of SP. They constructed a reference quality assembly for LA2093 and compared this to the well used Heinz accession. Furthermore, they utilized the genotyping approach of the so identified SV to learn more about their potential impact on RNA expression and thus phenotypes across the cultivar. Overall the manuscript is well written and easy to follow. In the following I list my questions and concerns in no particular order:

1. I noticed you detect the SV based on a genomic alignment. How do you handle the heterozygous SV or the detection of their genotypes? This can become a problem as given the assembly alone you will miss multiple SV and you seem to rely on the assembly.
Response: We agree with the reviewer that SV detection based on genome alignment will miss heterozygous SVs. However, heterozygosity will be a minor issue as both LA2093 and Heinz 1706 are highly inbred lines. We confirmed this by genotyping the identified SVs in LA2093 and Heinz 1706 using the Illumina reads and found that only 0.38% and 0.46% of SVs are heterozygous in LA2093 and Heinz 1706, respectively (Please check our response to question 7 of this reviewer). It is worth noting that we did consider heterozygous SVs in the genotyping process of the tomato panel, as described in the Methods section (Line 521-525).

2. I am maybe a bit confused as you are also using a mapping approach but I am not clear how they were put together, but I think you used the mapping to only confirm the SV detected by assembly??
Response: The mapping-based SVs were mainly used to confirm the SVs detected by genome comparison. In addition, they were also used to convert some complex SVs, including repeat and tandem expansion/contraction, into simple indels, and to redefine the precise boundaries of these indels, as described in the Methods section (Line 495-497).

3. I noticed that you described the detection of insertions, deletions and inversions, but no duplications or translocations were detected. Was that by design? And if so why did you ignore these?
Response: Our SV detection largely followed the Assemblytics algorithm (Nattestad and Schatz, Bioinformatics 2016, 32:3021-3023), which reports six types of SVs, including insertion, deletion, repeat expansion, repeat contraction, tandem expansion and tandem contraction. Repeat and tandem expansions/contractions are insertions/deletions taking place within tandem and repeat sequences, respectively. Therefore, in our study, we converted the repeat and tandem expansions/contractions to indels with precise boundaries so these SVs can be accurately genotyped in the tomato accessions using short reads.

4. I would suggest to redefine indel vs. SV sizes. Typically indels (2bp -50bp) are much smaller than SV (50bp+). That would make it later in the text easier to distinguish.
Response: While most studies define the size of SVs as >50 bp, this is arbitrary. For example, a recent study (Alonge et al., *Cell* 2020, 182:145-161) defined the SV size as >30 bp. To simplify

the description of the indels and inversions (all >10 bp), we prefer not to break them into two categories suggested by the reviewer.

5. Did you observed the standard length distributions across the indels?
Response: Yes, the length distribution of indels in our study is similar to that reported in other studies (e.g., Alonge et al., *Cell* 2020, 182:145-161).

6. It is surprising that 21,875 and 19,590 of the predicted genes were impacted by at least one indel. Is that due to the assembly strategy or the lack of improving the accuracy? I somehow doubt that it is a biological signal as this would be very disruptive. Are these indels in introns vs. exons?
Response: We thank the reviewer for bringing this up. The widespread nature of SVs related to the structure or presence/absence of genes has been revealed in several recent studies based on whole genome comparison in plants, such as tomato (Alonge et al., *Cell* 2020, 182:145-161), soybean (Liu et al., *Cell* 2020, 182:162-176), *Brassica napus* (Song et al., *Nat Plants* 2020, 6:34-45), grape (Zhou et al., *Nat Plants* 2019, 5:965-979) and rice (Zhao et al., *Nat Genet* 2018, 50:278-284). Alonge et al. (*Cell* 2020, 182:145-161) found that 95% of annotated genes in the Heinz 1706 genome have at least one SVs withing 5kb of coding sequences across 100 genomes.

The majority of gene-related SVs in our study are in the promoter, intron or UTR regions, with only ~2000 genes having SVs overlapping with the CDS regions. We have made this clear in the revised manuscript (Line 132-134; Supplementary Fig. 6).

7. I noticed that you implemented your own SV genotyping approach and confirmed that using ONT for one sample. I would encourage you also to report how many SV you found in addition to the genotyped SV for that comparison. Also I might have overlooked it but I don't see a description in the methods how the ONT reads were processed and SV were called.
Response: The description of the SV calling method using ONT reads has been added in the revised manuscript (Line 527-533). Thanks.

Regarding reporting the number of SVs identified using ONT reads but not by the short Illumina reads, we don't think this is necessary as we can expect that some additional SVs would be captured by long reads. Nonetheless, as suggested by Reviewer 3 (please check Comment 4 of Reviewer 3), we performed SV genotyping in the two reference accessions (LA2093 and Heinz 1706) using the Illumina short reads. We found that ~20% of SVs were not able to be genotyped in each of these two accessions. However, this is not unexpected as SV genotyping using short reads has high false negative rates, as reported in a number of studies (e.g., https://www.nature.com/articles/s41467-019-11146-4). This analysis further supported the high accuracy of SV genotyping (~98%) in our study. We have added this result (Supplementary Table 10) and a related sentence (Line 156-157) in the revised manuscript.

8. Maybe I missed it but when you are talking about FUL1+2 can you maybe describe the deletions and insertions more. Are they frameshifts, are they in their exons? Like you do it for SUCR. Furthermore, I noticed that for some of these listed events for certain genes you list the population frequency and for some not. Would be interesting to make that more consistent as these are important events.

Response: We thank the reviewer for pointing these out. We have made deletions and insertions in FUL1+2 clear (Line 243 and 250-252) and added population frequency for all described SVs in the revised manuscript (Line 252-254, 260-262 and 291-292).

9. I noticed you have specified the versions of some tools (e.g. Canu) but left that out for many. Please add this as this improves reproducibility of your study.
Response: Done. Thanks.

10. Are the scripts etc. available? I didn't see these referenced in the main text or methods. As you seem to have implement a lot of your own procedures/methods yourself (e.g. SV genotyping) are these programs available and benchmarked? Please make these public available etc.
Response: We have deposited the SV detection and genotyping scripts in GitHub and added a Code Availability section in the revised manuscript (Line 627-629).

Reviewer #3

Tomato is an important model for studying how eukaryotic genome structure influences genome function. The manuscript by Wang and Gao et al. is a major addition to this field, and will be of broad interest to the research community. Overall I find the work to be thoughtful, valid, and methodologically sound. I also find the conclusions to be reasonable given the data and analysis available. The paper presents a high-quality and curated *S. pimpinellifolium* reference genome as an important resource for the Solanaceae community. I believe this assembly and associated annotations will have much utility for a large number of researchers/projects.

However, some key findings are not novel/original because they were first described in the recently published paper (Alonge and Wang et al. Cell, 2020), a reference to which is missing from the manuscript. However, some findings are still novel and of great interest, including the finding of many candidate mutations associated with known genes underlying important traits. Additionally, the eQTL analysis identifying putative regulatory hubs presents major findings. Though the manuscript gives promising results regarding variants associated with important genes, the manuscript lacks functional genetics analysis to go beyond statistical association and directly assess the functional impact of candidate QTL.

Altogether, I commend the authors for their work. Please see below for more detailed comments as well as additional minor points that I believe would improve the clarity and impact of the manuscript.

Major Comments

Comment 1:
The manuscript omits some necessary references and/or descriptions of previous work. Firstly, the authors state that "draft genome assemblies of two accessions, LA1589 (ref. 4) and LA0480 (ref. 5), have been generated using Illumina short-read sequencing technology. Although these assemblies provided some essential genomic information for SP, they are highly fragmented and incomplete,". However, a chromosome-scale long-read SP assembly (accession BGV006775) was

published in Alonge et. al. 2019 (ref. 52). This should be included in the description of previous SP genome assemblies. This assembly is likely of lower base-accuracy and did not undergo de novo scaffolding, so the new LA2093 assembly is probably superior. Still, the previous assembly should be mentioned and cited in the introduction, and some comparisons should be made.
Response: We thank the reviewer for pointing this out. The assembly of BGV006775 has been mentioned and cited in the introduction (Line 44-46) and comparisons have been made (Line 89; Supplementary Tables S1 and S2). Indeed, the QV score of the LA2093 assembly is much higher than that of the BGV006775 assembly (46 vs. 28; Supplementary Table 2).

Secondly, Alonge and Wang et al., Cell, 2020 was recently published that is highly related to this manuscript but is not referenced. This publication must be cited and described in the introduction and in parts of the results section. For example, the sentence "However, the distribution of SVs in tomato accessions and their population dynamics are largely unexplored." is no longer true. Also, Alonge and Wang et al. Cell, 2020 describes sb1, another "suppressor of branching" QTL, and this should be mentioned along with sb3 in the introduction of known SVs influencing traits.

The Alonge and Wang et al 2020 publication renders some of Wang and Gao et al. as not novel. Please see comment 2 for more details. However, much of the work is still novel and has not been done before in tomato, such as the inversion analysis or the eQTL analysis. Furthermore, Alonge and Wang et al 2020 only examined 100 accessions with long-reads and did not genotype SVs in a larger set of accessions with short-reads. Wang and Gao et al. also provide valuable results about new potential variants underlying important traits, though functional validation is lacking (see comment 7).
Response: We have cited Alonge et al. 2020 and mentioned sb1 in the revised manuscript (Line 52 and 54). Thanks.

We are sorry for not citing this important paper in our original manuscript. We finished preparing the manuscript right before Alonge and Wang et al was published online and submitted the manuscript at around the same time when Alonge and Wang et al was published.

Comment 2:
I believe the introgression analysis (Figure 2 and S9) is not original/novel since these introgressions and others were described in great detail in Alonge and Wang et al 2020. At a minimum the authors should refer to these results as being previously described by Alonge and Wang et al 2020.
Response: We agree with the reviewer that findings from the introgression analysis are largely the same as those reported in Alonge and Wang et al. We have added one sentence citing Alonge and Wang et al in the revised manuscript (Line 202-203) and moved the related figures (Fig. 2e-g) to the Supplementary Figures section (Supplementary Fig. 9).

Comment 3:
Using Hi-C data, the authors show compelling evidence that the LA2093 inversions are real. However, I have a few comments regarding the analysis of diversity and recombination in the inverted regions. Firstly, what is meant by "diversity" in the results text and especially in Figure S6. This should be defined in the methods and caption, respectively. Secondly, their rationale for reduced recombination in the chr1 inversion region is unclear to me. The authors state that

“restriction of recombination was detected around the tomato fruit weight QTL, *fw1.1*”. However, LA2093 was not used as a parent in the *fw1.1* mapping populations. Furthermore, the referenced *fw1.1* paper states that 2/3 of chromosome 1 experienced reduced recombination, while the inversion described in this manuscript is only 14 Mbp long. The authors state in the introduction that LA2093 is a parent in a widely used RIL population. Therefore, can they use this data from this RIL population to assess recombination rates in the inverted regions? Broadly, if the authors intend to discuss reduced recombination rates, the conclusions should be much more clear.
Response: We thank the reviewer for point these out. Indeed, “diversity” in our original manuscript was not properly used. We have deleted this sentence and Figure S6 as we think this finding does not provide much novel information.

We agree with the reviewer that using the detected recombination restriction around *fw1.1* to support inversion-caused recombination restriction is not convincing. We have therefore deleted this part.

We did find that on the genetic map constructed from the widely used RIL population of LA2093 ×NC EBR-1, there is limited recombination in the inverted regions, as shown in Supplementary Fig. 3. However, some other non-inverted regions also show limited recombination. Therefore, we decided not to discuss the recombination restriction possibly caused by inversions as we think this is not strongly supported by the data we have.

Comment 4:
The authors forgo published short-read SV genotyping tools such as Paragraph (Chen et al, Genome Biology, 2019) and vg (Hickey et al, Genome Biology, 2020) and instead use a custom method. The authors provide evidence that the pipeline yields high specificity with their comparison of LA1589 long-read based SV calls. However, their analysis does not measure the sensitivity of their SV genotyping method. The authors state that 96.7% of the LA1589 genotyped SVs were confirmed with long-read LA1589 SV calling. But of the long-read SVs shared between LA1589 and LA2093, what percentage of them were genotyped with short-read data? The authors should also consider genotyping LA2093 SVs with LA2093 short reads to measure sensitivity/specificity. This would provide sensitivity information in addition to the specificity measurements given in the manuscript. This is necessary because the authors are using an unpublished method to genotype SVs with short reads.
Response: We thank the reviewer for the suggestions. Please check our response to question 7 of Reviewer 2.

Regarding the two SV genotyping tools mentioned by the reviewer, both of them were published recently and after we finished almost all the data analyses described in the manuscript. Nonetheless, our evaluation (Line 151-157) strongly supported the high quality of the SV genotyping using the pipeline we have developed.

Comment 5:
I commend the authors for a professionally established reference genome. The authors properly screen for contamination and false duplications. They also provide gene and repeat annotations. The authors must also provide a QV estimate with a tool such as mercury (https://github.com/marbl/merqury). I also encourage the authors to measure how many “pan-

genome genes” (Gao et al. 2019) were annotated in LA2093. Finally, please ensure that each LA2093 gene has a mapping to its homolog in ITAG4.*. This should be encoded in the GFF file. The authors may consider generally comparing the LA2093 genes with ITAG4.*.
Response: We thank the reviewer for the suggestion. We have performed QV estimate using Mercury for both LA2093 and BGV006775 genome assemblies, which further supported the high quality of the LA2093 assembly (a QV score of 46 for the LA2093 genome versus 28 for the BGV006775 assembly). The results have been added in the revised manuscript (Line 86-88 and Supplementary Table 2).

We found that 35,281 of the 40,369 genes in the pan-genome were present in LA2093. However, we think this result does not provide much interesting information so we did not include it in the manuscript. The gff file has been updated to include the corresponding homologs in ITAG4.

Comment 6:
Throughout the manuscript, the authors use two variant sets, one with respect to SL4.0 and one with respect to LA2093. For each analysis, it should be stated which assembly is being used as the reference. For example, exactly which reference/variants are used to build the trees in Figure S8, or for the eQTL analysis? Relatedly, this statement was unclear: “homozygous LA2093 alleles of the SVs were prevalent, taking up an average of 58.4% of the SVs in each accession, while only 39.4% SVs were homozygous for the Heinz 1706 genotypes”. This analysis should clarify how the variants are defined with respect to a reference genome. If using SL4.0 as the reference, homozygous 1706 alleles are not “variants”. So are these percentages with respect to two different coordinates systems?
Response: We thank the reviewer for pointing these out. eQTL analysis was performed using the LA2093 genome as the reference, as indicated in the Methods (Line 567-574). Also as indicated in the Methods, “SVs in a particular accession were classified as the LA2093 genotype (same as in LA2093), the Heinz1706 genotype (same as in Heinz 1706), heterozygous (containing both LA2093 and Heinz1706 alleles), or undetermined (genotypes that could not be determined due to insufficient read mapping information)” (Line 521-525), both reference genomes were used for SV genotyping, and the genotyped SVs were used to construct the tree (Figure S8a). SNPs were called using the LA2093 genome as the reference (Line 534) and used to construct the tree (Figure S8b). Nonetheless, we have revised this sentence to make it clearer (Line 168-170).

Comment 7:
The section “SV selection associated with breeding traits” presents very exciting results and underscores how much there is to discover when considering structural variation in genotype to phenotype studies. It is disappointing that no functional genetics was used to demonstrate causality for some of the putative variants underlying breeding traits. Still, the results are impactful. For most of the candidate variants, increasing (with domestication/modernization) allele frequency was paired with differential expression analysis to implicate candidate variants. Why was a differential expression analysis not performed for the Lin5 or AgpL1 examples? Also, what exactly is being shown in Figure S10A? Do the boxplots describe the distribution over biological replicates? It is confusing since this panel is only comparing two genotypes. And why is this the only expression analysis that uses RPKM rather than FPKM? This is not described in the methods -- all RNA-seq analysis and associated statistical (e.g. Figure S10, S11, and S14) methods should be described in the methods.

Response: We thank the reviewer for the positive comment that our results are impactful. We also agree with the reviewer that functional characterization of some candidate variants would provide critical insights into genetic and molecular mechanisms underlying the regulation of the traits; however, we believe this is beyond the scope of this study and will be pursued in our future studies.

The 15-bp deletion in *Lin5* is in its CDS, causing potential functional changes (Line 285-287). Differential expression analysis was not performed for *Lin5* as it is not relevant here. For *AgpL1*, based on RNA-Seq data of fruit development in LA2093 and NC EBR-1, the gene was very lowly expressed after the breaker stage (mostly <1 FPKM), while expressed at a much higher level in fruits at the mature green stage in both LA2093 (FPKM of 16.4) and NC EBR-1 (FPKM of 4.9). The expression data of the 399 accessions used in our study was derived from fruits at the orange stage (Zhu et al., Cell 2018, 172:249-261), at which *AgpL1* was lowly expressed (an average of 0.35 FPKM). Therefore, we did not present expression in accessions with or without the two mentioned deletions related to *AgpL1* as we think the expression data was not from the desired fruit stage.

Fig. S10A shows the expression from three or four biological replicates. We have made this clear in the legend. RNA-Seq expression profiles used for Figure S10A were derived from single-end reads, for which RPKM is equivalent to FPKM. Nonetheless, we have changed "RPKM" to "FPKM" in Figure S10A to make it consistent.

As indicated in Figure S10, S11 and S14, t test was used to test the significance of expression difference. RNA-Seq data processing and analysis methods have been updated in the revised manuscript (Line 567-573).

Minor Comments

Comment 8:
On multiple occasions, the authors use the word "significant" without providing the statistical rationale (such as a p-value) inline. Many of the p-values and statistical tests are described in the methods, but they should also be presented in parentheses after sentences in the results section.
Response: We have added corresponding p values when necessary. Thanks.

Comment 9:
Figures 2G and S9 are not interpretable given the caption. The caption should give some description of how to interpret the figure and can refer to the methods for more details.
Response: Done. Thanks.

Comment 10:
"... SP has been used as an important germplasm donor in modern breeding of tomato". This should be changed to "... SP has been used as an important germplasm donor in modern tomato breeding".
Response: Done. Thanks.

Comment 11:

I recommend not using the term “high-confidence” when describing the variant calls. This is appropriate when there is a counterpart set (like “low confidence”, e.g. wheat reference gene models).
Response: “high-confidence SVs” and “high-confidence SNPs” are widely used in literature (e.g., https://www.nature.com/articles/s41586-020-2371-0). Therefore, we kept it in the manuscript.

Comment 12:
“Genotyping these SVs in ~600 representative tomato accessions unravels alleles” - The word “unravels” is not appropriate here.
Response: Changed to “identifies”. Thanks.

Comment 13:
“discovers numerous novel SVs underlying genes known to regulate important breeding traits such as fruit weight and lycopene content” - this should be rephrased since an SV cannot “underly” a gene.
Response: Changed to “overlapping”. Thanks.

Comment 14:
The term “master regulator” is conventionally used for pioneer transcription factors. The authors should consider if their use of this term is appropriate and/or use an alternative description.
Response: We think the use of “master regulator” in our manuscript is appropriate. “master regulator” has been widely used in eQTL studies and can be used not only for transcription factors, but also for other types of proteins (e.g., protein kinases, https://scholar.google.com/scholar?q=master+regulator+protein+kinase).

Comment 15:
Black arrows for figure S5 should indicate a “start” and “stop” (or similar). The authors align PacBio reads to confirm the inversions. I suggest also aligning LA2093 reads to the LA2093 assembly to show that alignments are not discordant at inversion breakpoints.
Response: Done as suggested. Thanks.

Comment 16:
“SVs in gene body and promoter regions can directly impact gene functions and expression” - This should be rephrased to consider both direct and indirect impacts.
Response: Done. Thanks.

Comment 17:
“Only 14.8% of identified indels overlapped with gene body and promoter” - rephrase as “gene body or promoter”
Response: Done. Thanks.

Comment 18:
Figure S8 should define what “k” is in the caption.
Response: Done. Thanks.

Comment 19:

The methods used to generate Figure S3 are not described
Response: Figure S3 shows the synteny between the assembly and a previously published genetic map constructed from the RIL population of LA2093 × NC EBR-1. The method used to generate the synteny has been added in the revised manuscript (Line 445-446).

Comment 20:
"SVs were prevalent, taking up an average of 58.4% of the SVs in each accession" - This sentence is unclear (also see comment 6).
Response: Thanks for pointing this out. We have revised this sentence to make it clearer.

Comment 21:
Following conventions in the literature, "cis" and "trans" should be used instead of "local" and "distant" eQTL.
Response: Done. Thanks.

Comment 22:
"eQTLs were located near the start and end of coding regions" - rephrase as "start or end"
Response: Done. Thanks.

Comment 23:
"Hi-C data and synteny with the Heinz1706 genome were integrated to construct the pseudomolecules." - The authors should be more specific about this process. Exactly how was the Hi-C and synteny information combined? Also, please provide the assembly statistics for the initial SALSA scaffolds.
Response: Thanks for the suggestion. We have made the pseudomolecule construction process clear in the Method section (Line 434-449). We don't think the assembly statistics for the initial SALSA scaffolds provide important information as SALSA scaffolding is an intermediate step, so we did not include them in the revised manuscript. However, if the editor and the reviewer still think that these statistics should be included, we will be happy to comply.

Comment 24:
The discussion is a narrow summary of the results. The authors should expand the discussion to describe the broad implications of this work. The authors should also emphasize the importance of tomato as a model, either in the discussion or the introduction, since many readers may not know that some of the leading work in this field is in tomato.
Response: Thanks for the suggestion. The Introduction and Discussion sections have been updated.

Reviewer #1 (Remarks to the Author):

Thanks to have answer and taken into account all the reviewers recommendations

Reviewer #2 (Remarks to the Author):

I would like to thank the authors for addressing all my questions and concerns and include the additional information into the main text.

Reviewer #3 (Remarks to the Author):

Overall, the authors addressed my concerns and have delivered an improved manuscript. The authors added a comparison with the BGV006775 assembly and they provided the necessary QV information for their new assembly. The authors also added an analysis measuring the specificity of their SV genotyper. Figures, captions, and methods have also generally been made more clear. For example, Figure S5 is now more clear and compelling. I only have a few minor comments:

Short-read genotyping: The word "reliability" should be replaced with "specificity" on line 156. While the relatively poor sensitivity may have been described in previous papers, the readers of this paper may not be familiar with that information and therefore may draw the wrong conclusions about the sensitivity of short-read genotyping. And "specificity" is well-defined while "reliability" is not.

Multi-reference analysis: It is good that the use of two references is clear in the methods, but you may consider making it a little bit more obvious at the beginning of the results section as well. Most readers are used to single reference analysis, so they may be confused. Also, on line 168, it states "taking up an average 169 of 58.4% of the SVs in each accession". I suggest replacing "taking" with "making".

"High-confidence": Though it is not surprising that other papers have used this term in the past, I still believe that it is bad scientific practice. Unless the term "high-confidence" has a specific quantitative or statistical meaning, it should be avoided. In contrast, it is justified to call the new genome assembly "high-quality" since multiple qualitative and quantitative measurements (QV, BUSCO etc.) support this description.

The data is not yet public on NCBI

Reviewer #1
Thanks to have answer and taken into account all the reviewers recommendations
Response: Thanks.

Reviewer #2
I would like to thank the authors for addressing all my questions and concerns and include the additional information into the main text.
Response: Thanks.

Reviewer #3
Overall, the authors addressed my concerns and have delivered an improved manuscript. The authors added a comparison with the BGV006775 assembly and they provided the necessary QV information for their new assembly. The authors also added an analysis measuring the specificity of their SV genotyper. Figures, captions, and methods have also generally been made more clear. For example, Figure S5 is now more clear and compelling. I only have a few minor comments:
Response: Thanks.

Short-read genotyping: The word “reliability” should be replaced with “specificity” on line 156. While the relatively poor sensitivity may have been described in previous papers, the readers of this paper may not be familiar with that information and therefore may draw the wrong conclusions about the sensitivity of short-read genotyping. And “specificity” is well-defined while “reliability” is not.
Response: We have replaced “reliability” with “specificity”.

Multi-reference analysis: It is good that the use of two references is clear in the methods, but you may consider making it a little bit more obvious at the beginning of the results section as well. Most readers are used to single reference analysis, so they may be confused. Also, on line 168, it states “taking up an average 169 of 58.4% of the SVs in each accession”. I suggest replacing “taking” with “making”.
Response: In the Results section, we already mentioned that the SVs “were detected by direct comparison of the two high-quality assemblies” or something similar in several places. “taking” has been replaced with “making”.

“High-confidence”: Though it is not surprising that other papers have used this term in the past, I still believe that it is bad scientific practice. Unless the term “high-confidence” has a specific quantitative or statistical meaning, it should be avoided. In contrast, it is justified to call the new genome assembly “high-quality” since multiple qualitative and quantitative measurements (QV, BUSCO etc.) support this description.
Response: We have removed “high-confidence” in the manuscript.

The data is not yet public on NCBI
Response: The data is now public.
